# Production and Processing of siRNA Precursor Transcripts from the Highly Repetitive Maize Genome

**DOI:** 10.1371/journal.pgen.1000598

**Published:** 2009-08-14

**Authors:** Christopher J. Hale, Karl F. Erhard, Damon Lisch, Jay B. Hollick

**Affiliations:** Department of Plant and Microbial Biology, University of California Berkeley, Berkeley, California, United States of America; The University of North Carolina at Chapel Hill, United States of America

## Abstract

Mutations affecting the maintenance of heritable epigenetic states in maize identify multiple RNA–directed DNA methylation (RdDM) factors including RMR1, a novel member of a plant-specific clade of Snf2-related proteins. Here we show that RMR1 is necessary for the accumulation of a majority of 24 nt small RNAs, including those derived from Long-Terminal Repeat (LTR) retrotransposons, the most common repetitive feature in the maize genome. A genetic analysis of DNA transposon repression indicates that RMR1 acts upstream of the RNA–dependent RNA polymerase, RDR2 (MOP1). Surprisingly, we show that non-polyadenylated transcripts from a sampling of LTR retrotransposons are lost in both *rmr1* and *rdr2* mutants. In contrast, plants deficient for RNA Polymerase IV (Pol IV) function show an increase in polyadenylated LTR RNA transcripts. These findings support a model in which Pol IV functions independently of the small RNA accumulation facilitated by RMR1 and RDR2 and support that a loss of Pol IV leads to RNA Polymerase II–based transcription. Additionally, the lack of changes in general genome homeostasis in *rmr1* mutants, despite the global loss of 24 nt small RNAs, challenges the perceived roles of siRNAs in maintaining functional heterochromatin in the genomes of outcrossing grass species.

## Author Summary

Most eukaryotic genomes are divided into two functional classes of regulation: the euchromatic and the heterochromatic. Heterochromatic regions, often composed of potentially deleterious transposons and retrotransposons, are typically viewed as “silent” or not transcribed. Paradoxically, evidence from multiple organisms indicates that heterochromatic regions must be transcribed to maintain a heterochromatic character. In plants, specialized RNA polymerase complexes are thought to specifically process repetitive regions of the genome into small RNA molecules that facilitate maintenance of a heterochromatic environment. We investigated the role of this specialized polymerase pathway in maintaining maize genome homeostasis with particular focus on RMR1, a novel protein related to a family of DNA repair proteins, whose function in modifying repetitive regions of the genome is unknown. We find most small RNA generation is dependent on RMR1, which appears to function downstream of the specialized polymerase, RNA polymerase IV. However, we provide evidence that the function of RNA polymerase IV is not disrupted by the absence of small RNA generation. Our results suggest the division of the plant genome into euchromatin and heterochromatin is maintained by template competition between the specialized plant polymerases and canonical RNA polymerase II, and not by the subsequent generation of small RNA molecules.

## Introduction

A common feature of higher eukaryote genomes is an abundance of repetitive sequences, represented primarily by retroelements and DNA transposons. These repetitive sequences are often characterized as being heterochromatic, displaying both DNA and histone-level modifications associated with repressive chromatin environments [1]. Such repetitive regions are often over-represented in small RNA populations [2] and these small RNAs are thought to recruit chromatin modifiers that nucleate a repressive environment [3].

Repetitive sequences in plants are targeted by an RNA-directed DNA methylation (RdDM) pathway [4]. The protein effectors and sequence targets of RdDM are similar to the emerging piwiRNA pathway in metazoans and the siRNA heterochromatin pathway in *Schizosaccharomyces pombe* [3]. In *Arabidopsis*, a model for the RdDM pathway [4] proposes that aberrant RNA transcripts are generated by the activity of the plant-specific RNA polymerase IV (Pol IV) complex. These aberrant RNAs are processed into double stranded RNA via an RNA-dependent RNA polymerase (RDR2), and then cleaved into small interfering RNAs (siRNAs) approximately 24-nt in length via a *Dicer*-*like* protein, DCL3. The siRNAs associate with an Argonaute protein (AGO4) that interacts with the C-terminus of NRPE1, the largest subunit of a second plant-specific RNA polymerase known as Pol V. The Pol V complex transcribes genomic sequence targeted for RdDM and in so doing presumably tethers the AGO4 complex to target DNA sequences via small RNA-nascent RNA interactions [5],[6]. Downstream of the recruitment of this small RNA-containing complex, protein effectors of de novo DNA methylation and histone methylation are recruited [4],[7].

In *Arabidopsis* the function of RdDM remains enigmatic. Loss of certain RdDM pathway components abolishes most 24-nt RNA species [8]–[11]. Intriguingly, the loss of these small RNAs is not associated with any gross morphological defects, though some RdDM mutants are delayed in flowering time [12]. The paucity of morphological defects in RdDM mutants may be attributed to redundant mechanisms of heterochromatin maintenance [13],[14] or to the streamlined nature of the *Arabidopsis* genome. The *Arabidopsis* genome is composed of ∼10% repetitive sequence, most of which is found in pericentromeric regions [15]. Perhaps as a consequence of this genomic organization, very few *Arabidopsis* genes are close to small RNA clusters [10], and the expression patterns of correspondingly few genes are directly affected by RdDM mutations [16]. Recently it was proposed that the RdDM pathway may act as a backup mechanism for directing patterns of DNA methylation [17].

Multiple components of a maize RdDM pathway have been identified using genetic screens for factors necessary to maintain repressive states associated with paramutations. The *mediator of paramutation1* (*mop1*) locus was found to encode an ortholog of *Arabidopsis* RDR2 [18],[19], and the *required to maintain repression6* (*rmr6*) locus was recently shown to encode the ortholog of the largest subunit of the RNA polymerase IV complex, *Arabidopsis* NRPD1 [20]. For simplicity and uniformity in nomenclature we refer to the MOP1 and RMR6 proteins as RDR2 and RPD1 respectively. Previously we identified the maize *required to maintain repression1* (*rmr1*) locus as encoding a Snf2-like ATPase responsible for maintaining both specific cytosine methylation patterns at a DNA transposon fragment and its cognate ∼24 nt RNA species [21]. RMR1 is the founding member of a previously uncharacterized set of plant-specific proteins that shows similarity to two other groups of proteins defined by *Arabidopsis* DRD1 and CLSY1 [21].

While multiple loci encoding Snf2-like proteins have been identified in genetic screens for small RNA-directed silencing behaviors, the role of these ATPases in the RdDM pathway remains obscure. CLSY1 was identified in screens for components required for intercellular spreading of RNA-induced silencing [22]. DRD1 is required for some examples of *de novo* cytosine methylation [23],[24] and was recently shown to be necessary for Pol V associations with a DNA template and for subsequent transcriptional activity [5]. The role of the presumed *Arabidopsis* RMR1 ortholog remains unknown.

Here we show that RMR1 is responsible for the accumulation of a majority of maize 24 nt RNAs, and for the RDR2-independent inactivation of an autonomous *Mutator* DNA transposon. Additionally, both RMR1 and RDR2 are necessary for the accumulation of non-polyadenylated LTR retrotransposon RNA transcripts in a manner that is distinct from the role of Pol IV, which is necessary for the repression of polyadenylated transcripts from the same sampling of elements that are targeted by RMR1 and RDR2. These results point to an unexpected role for the RMR1 class of ATPases in mediating amplification of non-polyadenylated transcripts downstream of the repressive activity of Pol IV. Interestingly, we also find that in the highly repetitive maize genome, the loss of 24 nt RNAs is not associated with any obvious perturbation of genome homeostasis. On the contrary, the loss of RMR1 appears to dampen the phenotypic variances typical of inbreeding depression. This finding stands in contrast to results from plants deficient for maize RPD1 [25] and RDR2 [26] indicating that maintenance of the complex maize epigenome is dependent on mechanisms, including Pol IV function, that do not strictly correlate to accumulation of small RNAs.

## Results

### RMR1 function maintains the majority of 24 nt RNAs

**Figure 1:**
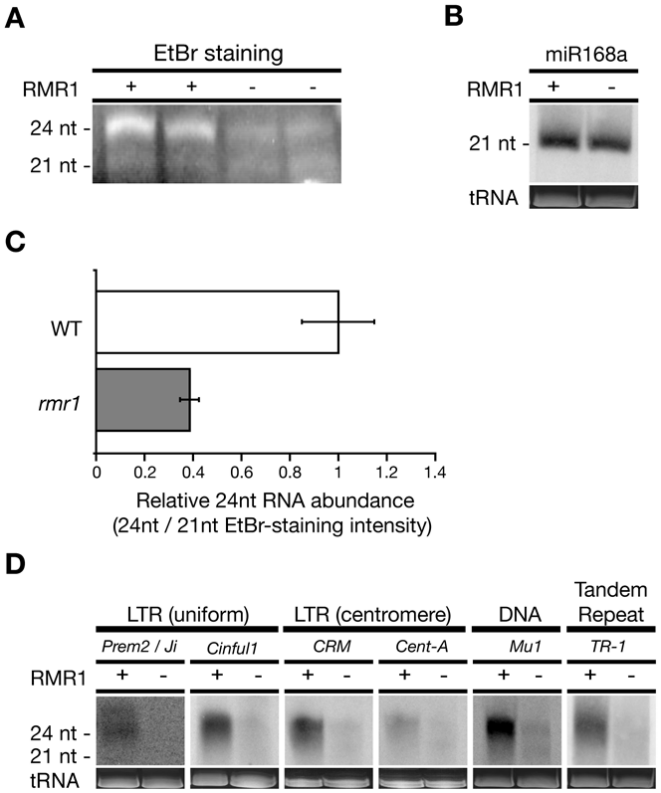
24 nt RNA populations are reduced in *rmr1* mutants. (A) EtBr staining of PAGE fractionated small RNAs from *rmr1-1* homozygotes (−) and *rmr1-1/+* siblings (+). (B) Small RNA northern blot hybridized with radiolabeled oligonucleotide complementary to miR168a. (C) Ratio of EtBr-staining intensity of the 24 nt RNA populations from *rmr1-1/+* (WT) and *rmr1-1/rmr1-1* (*rmr1*) genotypes standardized to the 21 nt RNA species (+/− 1 s.e.m.; adjusted so WT = 1). (D) Small RNA northern blots hybridized with radiolabeled riboprobes homologous to multiple repetitive elements found in the maize genome.

Using gel blot hybridization, we previously observed that ∼24 nt RNAs homologous to a CACTA-type DNA transposon directly upstream of the *Pl1-Rhoades* allele were lost in homozygous *rmr1-1* mutant plants [21]. Subsequently we found that we could resolve both 24 nt and 21 nt RNA populations on ethidium bromide (EtBr)-stained denaturing polyacrylamide gels [20]. Using this bulk level of analysis, the 24 nt RNAs were observed to be reduced in *rmr1-1* homozygotes relative to heterozygous siblings while the abundance of 21 nt RNAs appeared unchanged (Figure 1A). We confirmed that miR168, a representative of the 21 nt RNAs that are indicative of microRNAs (miRNAs) and trans-acting siRNAs [27], remained unaffected in mutants via small RNA northern blot (Figure 1B) [28]. Using the 21 nt size class as an internal reference, we compared the EtBr-staining intensities between *rmr1-1/rmr1-1* and *rmr1-1/+* genotypes and found that 24 nt RNAs accumulate to approximately 36% (two-sample z test; z = 2.37; p<0.05) of non-mutant levels in *rmr1-1* homozygotes (Figure 1C). These data are consistent with those obtained in our analysis of *rdr2* (*mop1-1*) mutants (Figure S1) and *rpd1* (*rmr6-1*) mutants [20], indicating that these results reflect a general disruption of RdDM-associated small RNA accumulation.

Small RNA northern analyses confirmed that the accumulations of small RNAs are lost in both *rmr1* and *rdr2* mutants (Figure 1D, Figure S1B) for a sampling of retrotransposons, DNA transposons, or tandem repeats, irrespective to the primary genomic localization of the cognate sequences. *CentA* and *CRM* LTR retrotransposons preferentially accumulate at the centromeres [29], whereas *Prem2/Ji* and *Cinful1* elements are found throughout heterochromatic and euchromatic regions [29]. Together with our previous report [21], RMR1 function appears necessary for the specific production of 24 nt RNAs representing repetitive sequences irrespective of element distribution or type. The dependence of small RNA accumulation on a Snf2-like protein is distinct from the results of *Arabidopsis drd1* mutants in which endogenous small RNA populations remain unaffected [30] but is reminiscent of the proposed function of the structurally distinct *Arabidopsis* CLSY1 [22].

### RMR1 affects inactivation of an autonomous DNA transposon in a manner distinct from RDR2

**Figure 2:**
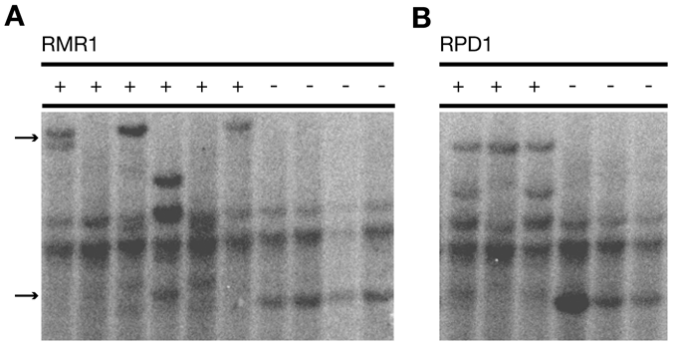
Southern blots of *Hin*fI-digested genomic DNA hybridized with a radiolabeled *Mu1* fragment. The upper arrow identifies high-molecular weight restriction fragments indicative of methylated *Mu1* and the lower arrow identifies hypomethylated *Mu1* fragments. (A) Blot for genomic DNAs isolated from *rmr1-1* homozygotes (−) and heterozygous siblings (+). (B) Blot for genomic DNAs isolated from *rpd1-1* (*rmr6-1*) homozygotes (−) and heterozygous siblings (+).

A hallmark of RdDM-pathway siRNAs is their correlation with increased cytosine methylation at target DNA sequences [4]. Cytosine methylation levels of a CHH context at a CACTA-like element directly upstream of the *Pl1-Rhoades* allele are reduced in *rmr1* mutants [21]. Similarly, RDR2 function is necessary to maintain cytosine methylation patterns at these same sequences, and at the terminal inverted repeats (TIRs) of *Mutator* transposons [21],[31]. Both *rmr1* and *rpd1* mutants show TIR hypomethylation of endogenous non-autonomous *Mutator* (*Mu1*) elements (Figure 2A and 2B) in line with previous results using *rdr2* mutants [31] and our *Mu1* small RNA analysis for *rmr1* mutants (Figure 1D). These results show that the default methylation status of these endogenous repetitive elements is affected similarly in all maize RdDM mutants examined to date.

Disparate roles for RDR2 have been shown in the establishment versus maintenance of repression at endogenous *Mutator* transposons by an inverted repeat of an autonomous *Mutator* transposon (*MuDR*) known as *Mu Killer* (*Muk*) [19],[32],[33]. *Muk* facilitates heritable hypermethylation of *MuDR* elements [34]. While RDR2 is necessary for the long-term heritable maintenance of *Muk*-induced repression [32], it is not necessary for the initiation of this repression. These observations are consistent with a model in which *Muk* hairpin RNA is processed to 24 nt effector RNAs independent of RDR2 action [33].

**Figure 3:**
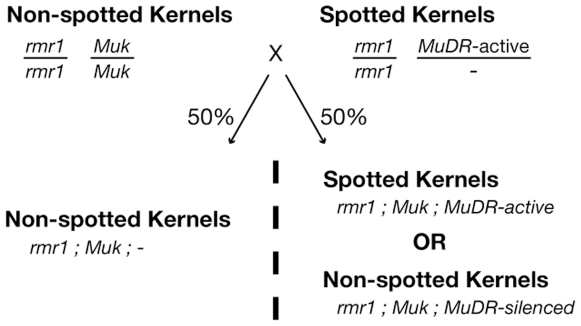
Crossing scheme used to assess role of RMR1 in establishing *Muk*-based silencing of *MuDR*.

To place RMR1 in an RdDM pathway relative to RDR2, we assessed the role of RMR1 function in the establishment of *Muk*-induced repression of *MuDR*. By crossing *rmr1-1* homozygotes having both an active *MuDR* element and a mutable color allele (*a1*-*mum2*) to *Muk* homozygous plants that are also homozygous for either functional *Rmr1* or the mutant *rmr1-1* allele (Figure 3) we were able to determine that, in contrast to RDR2, RMR1 is required for the initiation of *Muk*-induced *MuDR* repression. In this analysis, *MuDR* activity is reflected by a kernel-spotting phenotype due to somatic excisions of a *Mu1* element from the *a1*-*mum2* allele during kernel development [19]. Spotted kernels thus indicate an active *MuDR* element, while non-spotted or weakly spotted kernels indicate an inactive *MuDR* element (Figure S2). As expected, crosses of an *rmr1-1* homozygote carrying active *MuDR* to *Muk* plants that were homozygous for non-mutant *Rmr1*, produced few weakly spotted kernels (Table 1). However, when the same *rmr1-1* homozygote was crossed to *Muk* plants that were homozygous for the *rmr1-1* mutation, spotted kernels were recovered in the expected genetic ratios (Table 1, Figure S2) indicating *Muk*-repression of *MuDR* in the kernels was abrogated.

**Table 1:** Crosses between *Muk* homozygotes with or without functional RMR1 and *rmr1-1/rmr1-1 ; MuDR/-* pollen parents.

Female Genotype	Pollen Parent	Progeny Kernel Phenotypes		
		No spots	Spotted	Percent Spotted^1^
***Rmr1/Rmr1 ; Muk/Muk***	1	305	27	8%
	2	291	67	19%
	3	255	41	14%
	4	235	1	0%
	Total	1086	136	11%
***rmr1/rmr1 ; Muk/Muk***	1	48	46	49%
	2	38	43	53%
	3	157	94	37%
	4	60	39	39%
	Total	303	222	42%

1 Average spotting frequencies between two progeny sets are significantly different (P = 0.0009) using a Student's *t*-test.

The observation that initiation of DNA transposon silencing by an endogenous hairpin RNA is disrupted in *rmr1* mutants stands in contrast to the results from plants lacking RDR2 function in which similar initiation was unaffected [19]. Thus, while transposon-like sequences may be a common target of the maize RdDM pathway, the RMR1 and RDR2 proteins act differently with respect to their roles in mediating the type of trans-regulation induced by inverted repeats.

### RMR1 and RPD1 inversely affect the accumulation of LTR retrotransposon transcripts

Given that the RdDM pathway in both *Arabidopsis* and maize targets repetitive sequences primarily, we sought to characterize the response of other repetitive features in the maize genome to defects in RMR1 function. Because small RNAs functioning in RdDM are thought to transcriptionally repress targeted genomic regions, we expected that the loss of repetitive small RNAs in *rmr1* mutant plants would correlate with changes in transcript abundance derived from the respective sequences targeted in the genome. The maize genome is nearly 20-fold larger than that of *Arabidopsis* [35] due primarily to the expansion of LTR retrotransposon-derived sequence [36]. This preponderance of LTR retrotransposon sequences in the genome, coupled with the observation that RMR1 maintains small RNA populations corresponding to such sequences, indicated that these elements would be ideal targets for detecting bulk changes in transcript abundance that are dependent on RMR1 function.

**Figure 4:**
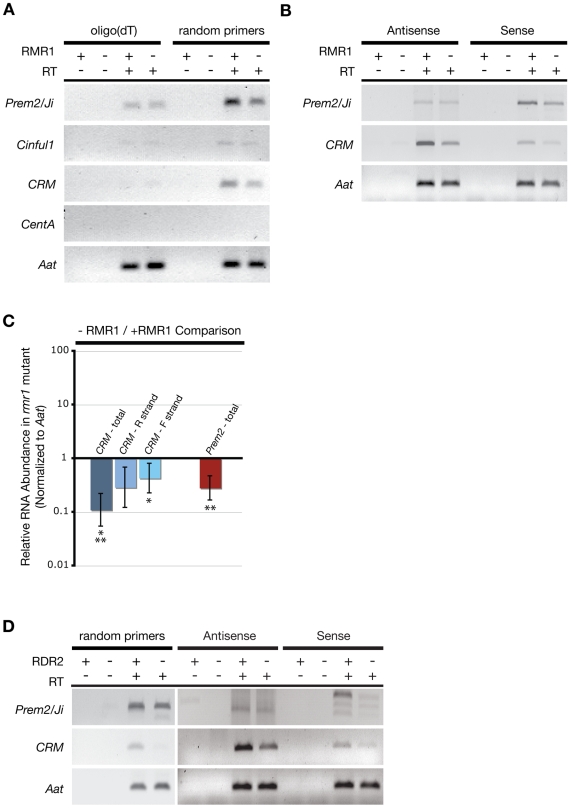
RT–PCR and qRT–PCR measurements of LTR transcripts in RdDM mutants. (A) Representative gel images of RT–PCR products for LTRs and the *Aat* control from both oligo(dT)-primed and random hexamer-primed cDNA for *rmr1* mutants (−) and non-mutants (+). (B) Expected size products of strand-specific RT–PCR. (C) qRT–PCR results comparing changes in abundance of LTR transcripts in *rmr1* mutants relative to non-mutants (+/− 2 s.e.m.). “*” and “**” denote significantly different paired results by two-sample z-test (p<0.05). (D) Representative RT–PCR products from random-primed cDNA, and strand-specific cDNA in *rdr2* (*mop1-1*) mutants (−) and non-mutant siblings (+).

Focusing on the same elements analyzed by our small RNA northern blots (Figure 1D), we performed reverse transcriptase (RT)-PCR over LTR regions (Figure S3) using cDNA synthesized from parallel B73 inbred lines with and without the *rmr1-1* mutation. Using oligo(dT)-primed cDNA, we detected little to no transcript for any of the LTRs, which is consistent with the observation that high copy number LTR retrotransposons are poorly represented in the polyadenylated RNA fraction [37]. To identify any potentially non-polyadenylated transcripts, we performed RT-PCR on random primed cDNA. Using this method we detected LTR transcripts in the non-mutant RNA populations (with the exception of *CentA*) indicating that, while these elements are transcribed, they are not polyadenylated (Figure 4A). Surprisingly, the abundance of these transcripts was reduced to varying degrees in *rmr1* mutants (Figure 4A). This result was unexpected as the canonical RdDM model posits that small RNAs and their derivative *de novo* cytosine methylation function to repress transcription of repetitive elements [4].

Strand-specific RT-PCR of the transcripts most readily amplified from random-primed cDNA (*Prem2/Ji* and *CRM*) indicated that both sense and antisense orientations of each LTR RNA transcript were present in non-mutant B73. In *rmr1* mutants both species of *CRM* transcript were reduced relative to non-mutant (Figure 4B). For *Prem2/Ji* we observed a loss of sense transcript but no apparent effects on the accumulation of antisense transcript in an *rmr1* mutant (Figure 4B). Additionally, we detected multiple *Prem2/Ji* antisense transcripts in our strand-specific RT-PCR experiments (Figure S4A) suggesting a diversity of these transcripts not present for the *CRM* transcripts. It is unclear why *Prem2/Ji* would show a difference in antisense RNA transcript regulation relative to *CRM* though it might be due to differences in genomic organization between these elements that will be more fully appreciated from the analysis of the completed maize genome. Importantly, the strand-specific RT-PCR demonstrated that for both *Prem2/Ji* and *CRM*, loss of RMR1 function correlated with loss of sense LTR transcript.

To quantify the loss of LTR RNA transcripts in an *rmr1* mutant, we performed quantitative RT-PCR (qRT-PCR) on the *CRM* and *Prem2/Ji* transcripts. The qRT-PCR results (Figure 4C) verified that these transcripts were significantly less abundant (>2 s.e.m. lower than non-mutant) in *rmr1* mutants, with *CRM* transcripts at 11% of non-mutant levels and *Prem2*/*Ji* transcripts at 28% of non-mutant levels as standardized to the *alanine aminotransferase* (*Aat*) internal control. Interestingly the levels of *CRM* RNA were more reduced in *rmr1* mutants as compared to *Prem2/Ji* (two-sample z-test; z = 2.16; p<0.05), consistent with the existence of *Prem2/Ji* RNAs unaffected by RMR1 function. Both the *CRM* and *Prem2/Ji* transcripts detected are relatively low abundance, with *CRM* found at levels at least 60-fold lower than *Aat* transcripts and *Prem2/Ji* at levels at least 15-fold lower than *Aat* (see Materials and Methods) despite the fact that both *CRM* and *Prem2/Ji* LTRs are estimated to be highly repeated in the genome, approximately 400 and 30,000 copies respectively [38],[39], relative to the single-copy *Aat*. Additionally, we performed qRT-PCR on the strand-specific cDNAs representing *CRM* in *rmr1* mutant and non-mutant plants to confirm that both sense and antisense strands are reduced in the *rmr1* mutants. Concordant with our semi-quantitative RT-PCR, we found that both strands accumulated to lower levels in *rmr1* mutants (Figure 4C). We also noted that the sense *CRM* transcripts were significantly less affected (two-sample z-test; z = 2.88; p<0.05) by loss of RMR1 function as compared to the overall reduction of *CRM* RNAs detected in the random-primed cDNA. This led us to conclude that, in contrast to *Prem2/Ji*, the centromeric *CRM* retrotransposons are preferentially represented by antisense transcripts that are stabilized by RMR1 function.

The loss of non-polyadenylated LTR transcripts in an RdDM mutant was unexpected, and stood in contrast to results in *Arabidopsis* in which levels of both the *At*SN1 non-LTR retrotransposon transcript and transcripts from sequences flanking a solo-LTR were elevated in *rdr2* mutants [27],[30]. In agreement with the results from *rmr1* mutants, we observed that both sense and antisense *CRM* transcripts were also reduced in *rdr2* mutants (Figure 4D). Total *Prem2/Ji* LTR transcripts were only slightly reduced, if at all, in *rdr2* mutants, though strand-specific RT-PCR of *Prem2/Ji* sense RNAs indicates that this strand is lost in *rdr2* mutants in a manner analogous to that observed in *rmr1* mutants (Figure 4D, Figure S4B). Intriguingly we observed a greater number of *Prem2*/*Ji* RNA species in both *rdr2* mutant and non-mutant siblings of an undefined genetic background than in the standard B73 line defining the *rmr1* mutant and non-mutant backgrounds (Figure S4A). While the accumulation of these RNAs responded to both the *rmr1* and *rdr2* mutations equivalently in the aggregate, these results indicate the potential diversity of LTR RNA dynamics between maize backgrounds [40], and highlight potential difficulties in making quantitative comparisons of repetitive RNA species between distinct genomic backgrounds. Correspondingly, qRT-PCR analysis of *CRM* transcripts confirmed a significant loss (>2 s.e.m. lower than non-mutant) in *rdr2* mutants relative to non-mutant siblings (Figure S4C), but because of the distinct genomic backgrounds we cannot be confident in comparing relative LTR RNA loss seen in the defined background of *rmr1* plants and the loss of RNA seen in the undefined background of our *rdr2* plants.

In total these results stand in contrast to observations of the regulation of solo-LTR and LTR-flanking sequence in *Arabidopsis rdr2* mutants [27],[30] and indicate that either RdDM function is distinct with regard to its effect on these specific LTR retrotransposons in the context of the maize genome, or that these LTR transcripts are differentially regulated from transcripts arising from LTR-flanking sequence. It is a distinct possibility that the loss of LTR transcript is accompanied with an increase in LTR-flanking transcripts as previously reported [30], but our analysis is of genomic averages of LTR transcripts and is thus unable to address this possibility.

**Figure 5:**
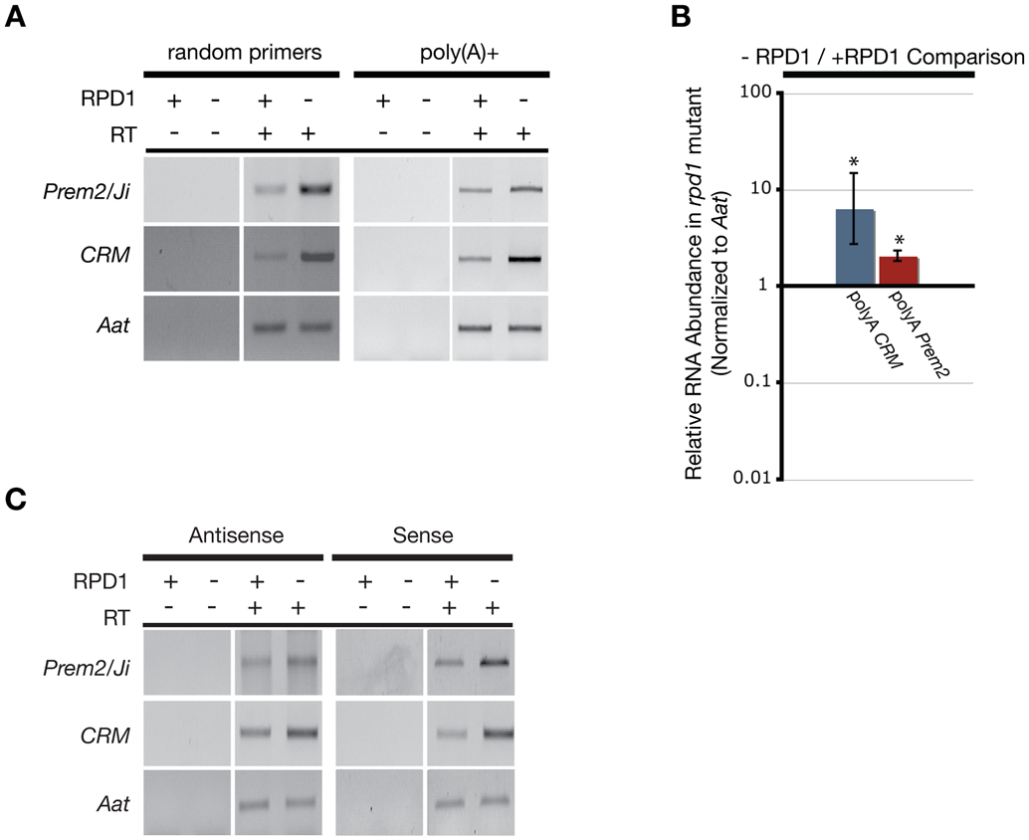
Loss of RPD1 function results in an increase in LTR RNA transcript levels. (A) Representative RT–PCR products amplified from random-primed cDNA from total and poly(A)-enriched RNA in *rpd1* (*rmr6-1*) mutants (−) and non-mutants (+). (B) qRT–PCR comparison of change in the relative abundance of polyadenylated *CRM* and *Prem2/Ji* in *rpd1* mutants relative to non-mutants (+/− 2 s.e.m.). “*” values are significantly different by a two-sample z-test (p<0.05). (C) Strand-specific RT–PCR products from *rpd1* mutants (−) and non-mutants (+).

We next looked at *Prem2/Ji* and *CRM* LTR transcript levels in *rpd1* (*rmr6-1*) mutants in a B73 genomic background equivalent to that of the *rmr1* mutants analyzed, as LTR retrotransposon representation in 24 nt RNA populations in *Arabidopsis* is dependent on Pol IV function [9]. In contrast to the results from *rmr1* and *rdr2* mutants, the absence of RPD1 function correlated with an increase in LTR transcript abundance (Figure 5A). Further, by fractionating total RNA populations using oligo(dT)-cellulose, we found that these increases in LTR RNA levels correlated with an increase of polyadenylated transcript (Figure 5A). We quantified both the efficiency of our poly(A) enrichment (Figure S5; Materials and Methods) and the increase of LTR transcript in the poly(A) fraction using qRT-PCR and found that *CRM* was ∼6-fold enriched and *Prem2/Ji* was ∼2-fold enriched in *rpd1* mutants as compared to non-mutant siblings (Figure 5B). In agreement with our *rmr1* quantitative data, *Prem2/Ji* transcript levels responded less to disruption of the RdDM pathway as compared to *CRM* (two-sample z-test; z = 2.63; p<0.05), though the level of *Prem2/Ji* transcripts still increased in the polyadenylated fraction. As Pol V transcripts were recently shown to lack polyadenylated tails [5], the likely source of the elevated *Prem2/Ji* and *CRM* polyadenylated transcripts is from RNA polymerase II. This is consistent to observations of *Arabidopsis nrpe1* mutants in which loss of Pol V at specific loci was accompanied by a reciprocal recruitment of Pol II complex members [5].

We confirmed that *CRM* and *Prem2/Ji* LTR RNA transcripts of both strand orientations are differentially affected in *rpd1* mutants as compared to *rmr1* mutants by performing strand-specific RT-PCR analogous to that carried out on *rmr1* and *rdr2* mutants (Figure 5C). Although we noted increases in all transcript abundances in the *rpd1* mutants, the *Prem2/Ji* antisense transcript appeared less affected. These results confirm the observation of our semi-quantitative analysis of *Prem2/Ji* and *CRM* transcript levels using random primed cDNA. The strand-specific RT-PCR results are also in agreement with the quantitative results showing an increase in polyadenylated *Prem2/Ji* and *CRM* RNA transcripts, transcripts that are presumably of the sense species for each element. The increased *CRM* antisense transcripts may represent either non-polyadenylated or polyadenylated RNA species.

### RMR1 is dispensable for genome homeostasis despite LTR retrotransposon-based expansion of the maize genome

**Figure 6:**
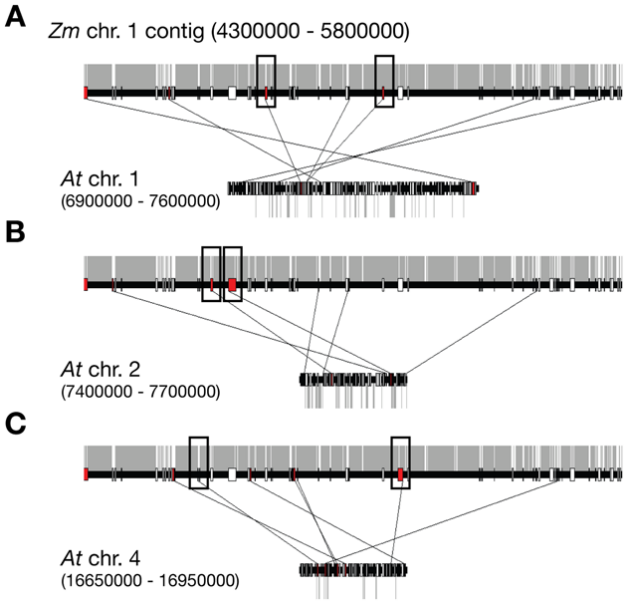
Synteny between regions of *Arabidopsis* and maize genomes illustrating increases in maize repetitive sequences. Comparisons between a 1.5 Mb section of the *Zea mays* (*Zm*) chromosome 1 contig and sections of *Arabidopsis* (*At*) chromosomes (A) 1, (B) 2, and (C) 4 with lines between regions identifying homologous gene pairs (red boxes). Gray bars above the maize and below the *Arabidopsis* chromosomal regions represent repetitive sequences. Boxed regions represent selected gene models analyzed in Figure S7, Figure S8, and Figure S9.

As RMR1 affects the RNA processing of at least a subset of LTR retrotransposons we sought to better understand the organization of the maize genome relative to *Arabidopsis* and the role of these repetitive elements in this genome expansion in order to gain insight into RMR1 function in the grass genome. We identified syntenic regions between two large sequenced regions of maize chromosomes 1 and 9 [41] and the *Arabidopsis* genome. Previous comparisons between *Arabidopsis* and another grass genome, rice, identified only a limited number of syntenous regions consisting of less than 20 genes per cluster [42] and we found similarly limited stretches of synteny consisting of clusters of usually 10 homologs or less between *Arabidopsis* and maize (Figure S6). One 1.5 Mb segment of the maize chromosome 1 supercontig was compared to segments of *Arabidopsis* chromosomes 1, 2, and 4 that contained syntenous homologs as identified by a BLASTP search (Figure 6). The maize sequence is relatively gene-poor as compared to the *Arabidopsis* regions, and the amount of repetitive sequence in the corresponding maize region is increased >10-fold relative to corresponding *Arabidopsis* sequence (gray bars in Figure 6, Table S1), primarily reflecting an increase of the LTR-class of retrotransposons (Table S1) [41]. These repetitive sequences have not only expanded in the intergenic regions, but have expanded into gene proximal and intragenic space of predicted gene models to a much greater extent in maize as compared to *Arabidopsis* (Table 2; Figure S7, Figure S8, Figure S9).

**Table 2:** Comparison of attendant repetitive sequence in *Zea mays* (*Zm*) and *Arabidopsis thaliana* (*At*) sequence.

Chromosomal Region^1^	Genes	Percentage of Genes with homology to repetitive sequence		
		500 bp upstream	Intragenic	500 bp downstream
*Zm* chromosome 1 contig (syntenous region)	63	11.11%	15.87%	19.05%
*At* chromosome 1 (syntenous region)	177	3.39%	2.26%	1.13%
*At* chromosome 2 (syntenous region)	73	4.11%	0.00%	4.11%
*At* chromosome 4 (syntenous region)	85	2.35%	1.18%	2.35%
*At* Totals (syntenous regions)	335	3.28%	1.49%	2.09%
*Zm* chromosome 1 contig (complete)	233	13.73%	20.17%	14.16%
*Zm* chromosome 9 contig (complete)	240	13.75%	19.58%	15.42%

1 Syntenous regions refer to the chromosomal and contig regions identified in Figure 6. Complete contig refers to the sequence generated by Bruggman et. al 2006 [41].

**Figure 7:**
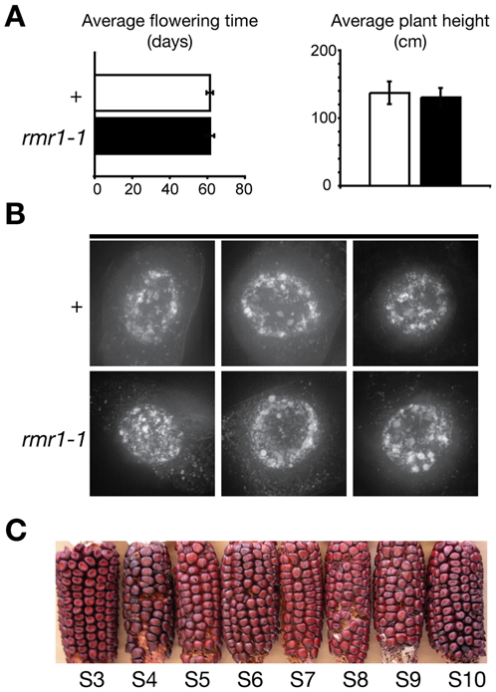
Plants deficient for RMR1 have no obvious morphological defects. (A) Comparison of average flowering times and plant heights between *rmr1-1* homozygotes (*rmr1-1*) and heterozygous siblings (+) (+/− 1 s.e.m.). (B) Deconvolution microscopy DAPI-stained nuclei isolated from growing root tips. (C) Self-pollinated ears of progressively inbred *rmr1-1* homozygotes from the 3^rd^ self-crossed generation (S3) to the 10^th^ (S10).

In both maize and *Arabidopsis*, epigenetic regulation of attendant repetitive sequences can affect gene expression [43],[44], and *Arabidopsis* siRNAs can presumably generate secondary RdDM proximal to an initial targeted locus [45]. However, the loss of 24 nt RNAs and LTR transcripts in *rmr1* mutants did not correlate with any gross morphological or sterility phenotype despite the expansion of repetitive sequences into genic regions. In three separate mutant allele backgrounds, *rmr1* mutations did not affect plant height or flowering time (Figure 7A, Figure S10A, S10B), and no obvious pollen sterility above background has ever been observed in *rmr1* mutants [21].

While *Arabidopsis* RdDM mutants do not show chromosome segregation defects [46], interphase heterochromatic foci are dispersed in *Arabidopsis drd1*, *nrpe1*, and *nrpd2* mutants [47],[48]. We observed 4′,6-diamidino-2-phenylindole, dihydrochloride (DAPI)-stained nuclei isolated from root tips of *rmr1-1* homozygous and *rmr1-1*/+ siblings under a UV light microscope (Figure S11A, S11B) and noted no changes in heterochomatic foci staining between mutant (n = 20) and non-mutant (n = 33) siblings. To better assess any potential changes in nuclear organization in *rmr1* mutants, we used deconvolution microscopy and again found no alterations in heterochromatic foci between mutant (n = 8) and non-mutant (n = 6) nuclei (Figure 7B). These results are similar to observations of *Arabidopsis nrpd1*, *rdr2*, *dcl3*, and *ago4* mutants which showed no significant changes in chromocenter organization [48].

The absence of morphological or large scale cytological defects in *rmr1* mutants, while consistent with prior analysis of multiple *Arabidopsis* RdDM pathway mutants, are surprising for a genome dominated by repetitive sequences. However, recent genome-wide expression profiling of *Arabidopsis* RdDM mutants indicates that, even among genes proximal to small RNA generating sequences, normal expression patterns persist in all but a small fraction of genes [16]. This is in line with RT-PCR analysis we carried out on a subset of maize genes (boxed genes in Figure 6, Figure S7, Figure S8, and Figure S9) that were identified with 23–24 nt small RNA signatures [49] within 1 kb upstream or downstream, or intragenic. These genes showed no RNA expression differences in *rmr1* mutants relative to non-mutants. Interestingly, the small RNAs representing repetitive sequences found in intragenic regions or directly downstream of predicted maize genes (Table 2) are lost to a lesser extent in *rdr2* mutants than those found upstream or in the genome as a whole (Table S2) [49]. This observation suggests the existence of small RNA processing pathways independent of RdDM that operate at repetitive sequences in gene proximal or intragenic contexts, and perhaps explains the paucity of effects of RdDM mutants on genic regions.

Most surprisingly from our pedigree analysis of *rmr1* mutants, we found we could successfully inbreed *rmr1-1* homozygotes by single-seed descent for 10 generations with little degradation in plant quality or seed set (Figure 7C). At the S_5_ generation of inbreeding, each of 4 homozygous *rmr1-1* lines showed uniformity of type with no obvious morphological defects and all plants (39/39) yielded normal seed set upon self pollination, whereas two lines derived from non-mutant F_2_ siblings (*Rmr1-A632* homozygotes) yielded only 55% (13/22) phenotypically normal plants at the S_5_ stage. Nine of 24 plants from the two *Rmr1-A632*/*Rmr1-A632* lines (3/9 and 6/13 off-types in the respective lines) were phenotypically abnormal. Four of 9 off-types were classified as “runts” (<∼1/3 of sibling height) and one of these had narrow leaf blades. None of these runts produced silking ears. Four plants had delayed silking relative to pollen shed; two of these plants produced tiny ears with no grains and two plants had normal sized ears with only a single grain each. One otherwise normal plant had vestigial apical leaves and no apical inflorescence. What was particularly intriguing about these observations is the uniformity of type in the homozygous *rmr1-1* lines, as the appearance of phenotypically variable plants seen in the non-mutant lines is typical of inbreeding depression. Thus, while RMR1 appears dispensable for genome homeostasis, it is possible the RMR1 function may be responsible for some trans-generational behaviors of the epigenome.

## Discussion

The Snf2 family of proteins that RMR1 belongs to encompasses a large group of functionally diverse proteins that are often referred to as “chromatin remodelers” based primarily on the functional analysis of *Saccharomyces cerevisiae* Snf2p [50]. It is unclear to what extent RMR1 might functionally overlap with Snf2p as a chromatin remodeling protein, if at all. RMR1, as well as *Arabidopsis* DRD1, and CLSY1 belong to a subfamily of Snf2 proteins defined by Rad54, an ATPase involved in homologous recombination (HR) via interactions with single-stranded and double-stranded DNA [51]. In plants it appears that a subgroup of these HR-related proteins has been co-opted for repression mechanisms involving the activities of plant-specific RNA polymerases.

**Figure 8:**
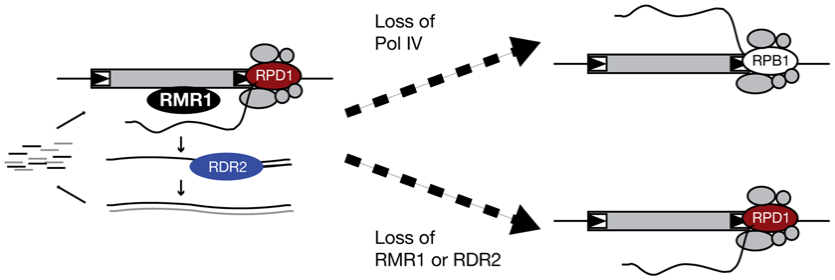
A model for RMR1, RDR2, and RPD1 action at LTR retrotransposons. Pol IV generates a low-level of precursor RNA that is amplified by RDR2 into double-stranded RNA that is then processed into small RNAs. The ability of Pol IV to generate a RDR2 template is facilitated by the action of RMR1. Upon loss of RPD1 the Pol IV complex fails to assemble on LTR retrotransposon templates and the elements are instead transcribed by the Pol II complex which generates polyadenylated transcripts. In the case of RMR1 or RDR2 deficiencies, the amplification of aberrant, non-coding transcripts by RDR2 is lost, but Pol IV still acts at LTR templates and prevents Pol II recruitment.

Our results with RdDM pathway mutants support a model in which RMR1 facilitates amplification of Pol IV-generated transcripts from repetitive sequences into small RNA precursor transcripts. These precursor transcripts are processed to small RNAs that feed back to homologous sequences in the genome, recruiting DNA methylation and maintaining repression of these elements (Figure 8). It seems likely that Pol IV is an active polymerase based on the recent findings in *Arabidopsis* that Pol V, a related polymerase, actively transcribes intergenic regions, and that both Pol IV and V have Pol II-like holoenzyme compositions including some Pol II components themselves [5],[52],[53]. Additionally, essential residues of the presumed Pol IV active site are necessary for normal function [54]. The role of RNA-dependent RNA polymerases in small RNA silencing pathways has long been held to be the amplification of dsRNA precursor molecules to biologically significant levels [55]. We postulate that the non-polyadenylated LTR transcripts detected in our samples are primarily RDR2-derived secondary transcripts, which would explain the apparent down-regulation of these RNAs in an *rdr2* mutant. The LTR retrotransposons analyzed here may be targets of Pol IV activity to the exclusion of Pol II even in the absence of RDR2, preventing generation of polyadenylated transcripts. Support for the effective targeting of Pol IV complex members independent of a functional RdDM pathway is found in the observation in *Arabidopsis rdr2* mutants that Pol IV nuclear localizations are unaffected [56]. The increase of *Prem2/Ji* and *CRM* transcript levels in *rpd1* mutants is likely due, in part, to the reciprocal gain of Pol II activity at Pol IV-targeted loci and the subsequent generation of transcripts that are no longer a substrate for RDR2 activity, and thus are not processed into small RNAs (Figure 8). This competition model between Pol IV and Pol II polymerases is supported by the shared subunit compositions of Pol IV and Pol V with Pol II [52].

We place RMR1 upstream of RDR2 activity and downstream of Pol IV based on the observation that RMR1 is necessary for the accumulation of sampled LTR RNA transcripts of both the sense and antisense orientations. As Rad54 proteins are known DNA-binding proteins [51], our model favors RMR1 acting at the DNA level, though an interaction with a nascent or sliced RNA analogous to the known homology search function of Rad54 is possible. In multiple biological systems Snf2 proteins have been implicated in small RNA-nascent RNA interactions [57],[58] and plant Rad54-like helicases may have been co-opted for such function. RMR1 function presumably facilitates the interaction between RDR2 and an extremely low-level Pol IV-derived RNA, though RMR1 does not appear to be a dedicated RDR2 complex member because our assays of *Muk*-induced *MuDR* repression indicate that *rmr1* mutants can affect silencing pathways that are unaffected in the *rdr2* (*mop1-1*) mutant. *Muk*-induced repression is proposed to take place through a long hairpin transcript precursor, structures known to be the target of a viral defense pathway in *Arabidopsis* [59], which utilizes the RDR6 RNA-dependent RNA polymerase in place of RDR2 [27]. Thus, RMR1 could act upstream of or in conjunction with multiple RNA-dependent RNA polymerases. This proposed role for RMR1 is distinct from the presumed function of the related *Arabidopsis* DRD1, but could potentially overlap or be fully analogous to the function of *Arabidopsis* CLSY1, which has similar effects on siRNA production [22], despite the fact RMR1 and CLSY1 are not direct orthologs [21].

It is curious that we were able to detect significant loss of non-polyadenylated LTR transcripts in *rmr1* and *rdr2* (*mop1-1*) mutants, while previous work had shown increase of *Arabidopsis* SINE element transcripts in *rdr2* mutants with functional Pol IV [5],[27]. It is likely due to the high LTR retrotransposon content of the maize genome described here and by others that we were able to directly amplify generic LTR sequence at all. Because our analysis of LTR transcripts and small RNAs represents a broad sampling of *Prem2/Ji* and *CRM* elements in the genome from a large number of distinct contexts, and as such only gives us an average of cumulative effects of RdDM mutations on these elements, it is possible that specific elements could be differentially affected.

Our results with *rmr1* mutants argue against the direct requirement of small RNAs themselves for proper genome maintenance. This is particularly intriguing in maize as targets of the RdDM pathway have expanded in overall number and proximity to coding regions, and because maize Pol IV mutant plants have developmental defects [20],[25]. The model described above may explain how *rpd1* and *rmr1* mutants could generate distinct phenotypic consequences. While *rmr1* mutants may lack a majority of small RNAs, our results with *Prem2/Ji* and *CRM* transcripts suggest that Pol IV function might remain engaged at repetitive genomic loci. In this way genome dysfunction might only occur when the Pol IV holoenzyme is lost, leading to misregulation of silenced genomic regions by recruitment of Pol II. The loss of Pol IV may also deplete Pol II holoenzyme at regions normally transcribed by Pol II, leading to stochastic defects in gene regulation. This could be particularly detrimental in a genome where a large amount of repetitive sequence might act to significantly titrate out Pol II complexes in the absence of Pol IV.

What remains an open question in the model put forth here and suggested elsewhere is the evolutionary significance of maintaining normal small RNA levels in the plant. This is particularly meaningful when viewed in light of the suggestion that Pol IV may function without other RdDM pathway members, or the recent finding that Pol V also has small RNA-independent functions in higher-order genomic organization [48]. Our observation of altered inbreeding behavior in *rmr1* mutants suggests that while small RNAs may not be required for normal development, they might have broad functions for out-crossing species such as maize that display regular and predictable differences in genome regulation depending on breeding strategies in ways that are not fully explained by genetic variation [60] and thus may have an epigenetic component.

## Materials and Methods

### Genetic stocks

All *rmr1*, *rpd1* (*rmr6*), and *rdr2* (*mop1*) mutations are described previously [18],[19],[21]. Materials used for *rmr1-1* and *rdr2* (*mop1-1*) small RNA, *rdr2* (*mop1-1*) RT-PCR, and *rmr1* morphological and *rmr1* cytological analyses are as previously described [21]. RT-PCR and qRT-PCR analysis comparisons between *rmr1* mutants and non-mutant plants were made using BC_3_F_2_ *rmr1-1* homozygotes (94% B73) derived from introgression of *rmr1-1* into a color-converted B73 inbred line and non-mutant plants from the parental B73 line (97% B73). For *rpd1* (*rmr6*), RT-PCR comparisons were made between homozygous mutants and non-mutant siblings from BC_3_F_2_ progeny (94% B73) derived from introgression of *rmr6-1* into the same color-converted B73 inbred as mentioned above. For the inbreeding analysis in Figure 7C, homozygous *rmr1-1* and homozygous *Rmr1-A632* recombinant inbred lines were established by single-seed descent from a specific A632-derived F_2_ progeny as previously described [61]. Growth conditions for plants can be found in Text S1.

### Small RNA abundance analysis

Small RNAs were enriched from total RNA extracted from 5 cm immature ear tissue and visualized following polyacrylamide gel electrophoresis (PAGE) as previously described [20]. After subtracting background staining intensity, the relative abundance of the ∼24 nt RNA population for each sample was calculated by standardizing the intensity relative to the densitometry value of the respective ∼21 nt small RNA fraction of the sample. This analysis was repeated for at least two technical replicates for each of four biological replicates for both *rmr1-1* homozygotes and heterozygous siblings. The relative abundances for each biological replicate were calculated from the averages of the technical replicates for each sample, and these averages were subsequently averaged themselves to represent the 24 nt RNA abundances for each genotype with the values represented relative to a non-mutant abundance = 1.

### Small RNA northern blots

All northern blots were carried out as previously described [21]. Probes used are listed in Table S3. The probes for retrotransposon LTRs were amplified by PCR from genomic DNA using primers previously described [62]. TR1 probe was amplified by PCR from genomic DNA using primers designed to a previously characterized tandem repeat [63]. All PCR amplicons were T/A cloned into the pGEM cloning vector (Promega), while the *Mu1* plasmid is as previously described [19]. Riboprobes for northern blot hybridization were generated as described [21]. A DNA oligonucleotide complimentary to maize microR168a [28] was end-labeled with gamma-32P- labeled ATP using polynucleotide kinase (Fermentas).

### Stock syntheses and analysis of *Mutator* activities

All *rmr1-1* materials were homozygous for the dominant *A1* allele needed for full pigmentation in both plant and kernel tissues. To generate *MuDR* lines homozygous for the *rmr1-1* allele, a single *rmr1-1* homozygote was first crossed to a plant carrying both an active *MuDR* element at a reference position known as *p1* and the *a1-mum2* reporter allele [19]. The resulting F_1_ progeny grown from fully pigmented kernels were self-pollinated to recover spotted kernels (*a1-mum2*/*a1-mum2*; *MuDR (p1)*) that were complemented for all other necessary kernel color factors. F_2_ progeny plants derived from these spotted kernels were genotyped for *rmr1* alleles as described [21] and *rmr1-1* homozygotes were used to establish inbred lines via single seed descent for four generations, each time selecting spotted kernels from ears segregating both spotted and non-spotted (no *MuDR*) kernels. The specific plants used as pollen parents for the *Muk* crosses were confirmed to be heterozygous for *MuDR (p1)* via the ∼1∶1 segregation of spotted and non-spotted kernels upon testcrosses to *a1-mum2*/*a1-mum2* plants (Figure S2). To generate the appropriate *Muk* stocks, a single *rmr1-1* homozygote was first crossed to a plant homozygous for both *Muk* and *a1-mum2*, and the resultant F_1_ progeny derived from fully-pigmented kernels were self-pollinated. Pale-colored F_2_ progeny kernels were selected to recover *a1-mum2* homozygotes [19]. A single F_2_ plant that was confirmed by genotyping to be homozygous for *Muk* and heterozygous for *rmr1-1*, [19],[21], was self-pollinated to generate the founding members of the *Muk ; rmr1-1/rmr1-1* and *Muk ; +/+* families. *MuDR* activity was assayed by visual inspection of kernel spotting following the crossing scheme outlined in Figure 3. Control crosses to non-mutant *a1-mum2/a1-mum2* plants were carried out in parallel to confirm the activity of *MuDR* as detailed in Table 1.

### Southern blot analysis

Genomic DNA samples from homozygous mutants and heterozygous sibling plants were digested with *Hin*fI restriction enzyme (New England Biolabs) and subjected to Southern blot hybridization with a *Mu1* radiolabelled probe as previously described [31].

### RT–PCR

RNA was isolated from seedlings 4 days post-imbibition via standard Trizol (Invitrogen) purification. Oligo(dT)-primed cDNA was generated as previously described [20]. Random primed cDNA was generated using 1 ug of total RNA that was reverse transcribed with the Superscript III enzyme (Invitrogen) in the presence of 250 pmol of random hexamers in a 20 µL reaction. LTR cDNA sequence was then amplified via PCR with previously described primers [62]. The *alanine aminotransferase* (*Aat*) cDNA was PCR amplified using previously described primers [20]. The PCR program used is as follows: 94°C for 30 sec, 57°C for 30 sec, and 72°C for 45 sec. This amplification cycle was repeated 35 times for *rmr1-1* and *rdr2* (*mop1-1*) cDNA analysis, and 30 times for the *rpd1* (*rmr6-1*) analysis. Strand-specific RT was carried out in the same fashion as the random-primed RT, except in the presence of 250 pmol of LTR-specific DNA oligonucleotides in the proper orientation, as well as DNA oligonucleotide primer specific to the *Aat* control RNA. PCR on strand-specific cDNA was carried out as described above.

### Quantitative RT–PCR analysis and transcript abundance calculations

Real time RT-PCR was carried out on random primed cDNA generated as described above. All reactions were performed on an ABI 7300 real-time cycler (Applied Biosystems) using the DyNAmo HS SYBR Green qPCR kit (New England Biolabs) following the manufacturer's instructions. Relative transcript abundances between *rmr1* mutant and non-mutant samples were calculated for *CRM* and *Prem2/Ji* from three technical replicates using the 2^-ΔΔC^_T_ method [64] with C_T_ values for 1 ng of starting cDNA normalized to C_T_ values of *Aat*. Quantifications of the strand-specific *CRM* transcripts were carried out similarly using independent biological samples and 2 technical replicates. Quantifications of *CRM* and *Prem2* between *rpd1* mutant and non-mutant siblings were carried out using three technical replicates and quantifications of *CRM* between *rdr2* mutant and non-mutant siblings were carried out using two biological replicates. Levels of *CRM* and *Prem2/Ji* transcripts relative to *Aat* transcript abundance were calculated by generating a standard curve of *Aat* levels based on a dilution series of starting cDNA amounts from 5 ng to 0.2 ng, this calculation assumes equal efficiencies of amplification for the control *Aat* products and the experimental *CRM* and *Prem2/Ji* products.

### Poly(A) fractionation of total RNA

For the selection of polyadenylated RNA from *rpd1* (*rmr6*-1) mutants (Figure 5A and 5B) ∼70 µg of total RNA was batch-purified using oligo(dT) cellulose (Ambion) as previously described (Cold Spring Harb. Protoc.; 2006; doi:10.1101/pdb.prot4047). As a positive control for the enrichment of the polyadenylated RNA fraction using this method we carried out qRT-PCR as described above comparing the relative levels of the polyadenylated *Aat* control transcript with the maize *45S* precursor transcript, which is not polyadenylated, in both the poly(A) enriched fraction and the flow-through. The *Aat* transcript was enriched >200-fold in both mutant and non-mutant samples relative to *45S* in the poly(A) fractions (Figure S5) indicating our fractionation was successful in enriching for polyadenylated transcripts.

### Cytological analysis

Root tips were collected from newly-germinated seedlings and fixed overnight in 3 parts 95% ethanol : 1 part glacial acetic acid. Following digestion with 2% (w/v) Onozuka R10 cellulase (Yakult Honsha), 1% (w/v) Macerozyme R10 (Yakult Honsha) as described [65], nuclei were fixed and mounted in acrylamide and DAPI-stained as previously described [66]. Microscopy was carried out on a DeltaVision imaging station (Applied Precision) and images were analyzed using DeltaVision/softWoRx software (Applied Precision) as described [66].

### Maize–*Arabidopsis* comparative analysis

Maize chromosome 1 and 9 sequence, and corresponding gene models are as previously described [41],[49]. Each predicted maize protein sequence was used to query the predicted protein sequences of the *Arabidopsis* genome (TAIR7 genome release) using BLASTP. The resulting BLASTP top *Arabidopsis* HSP for each predicted maize protein was used to construct a dot-plot comparing the location of homologous maize-*Arabidopsis* gene pairs along the length of their corresponding chromosome using DAGCHAINER [67]. The resultant dot-plots for the maize chromosome 1 sequence (Figure S6) were used to visually identify regions of maize-*Arabidopsis* synteny. The maize chromosome 9 contig did not appear to have any significant syntenic gene clusters with any *Arabidopsis* chromosome.

Using an in-house Perl script, we generated comparative maps between *Arabidopsis* and maize of the potentially syntenous regions identified in the visual dot-plot analysis (Figure S6). Repetitive sequence in these chromosomal regions, and the subsequently selected gene models (Figure S7, Figure S8, Figure S9) was identified using the CENSOR algorithm [68]. To generate the data in Table 2 the predicted gene models of both the *Arabidopsis* and maize chromosomal regions, as well as the 500 bp upstream and downstream sequence of each gene model, were used to query the Repbase database of repetitive elements (www.girinst.org) by a BLAST search. Those genes with significant homology to a repetitive element (E value<0e-5) were scored as having an attendant repetitive sequence upstream, downstream, or intragenic.

## Supporting Information

Figure S1 Plants deficient for RDR2 show analogous effects on small RNA populations as *rmr1* mutants. (A) EtBr staining of PAGE-separated small RNA fractions from *rdr2* mutants (−) and heterozygous siblings (+). (B,C) Small RNA northern blots hybridized with radiolabeled probes of various repetitive maize features and miR168 showing *rdr2* mutants specifically lose 24 nt small RNAs corresponding to repetitive sequence.
(0.32 MB TIF)

Figure S2 Representative ear progenies displaying active and silenced *MuDR* functions. (A) Test cross progeny of an *rmr1-1* homozygote with an active *MuDR* element (grey box) by a plant homozygous for the a1-mum2 reporter allele showing active *MuDR* (spotted kernels) segregates as expected from *rmr1* mutants. (B) Cross of parental *rmr1-1* homozygote from (A - grey box) to a *Muk* homozygote illustrating effective silencing of *MuDR* element. (C) Cross of parental *rmr1-1* homozygote from (A- gray box) to an *rmr1-1/rmr1-1* ; *Muk* plant showing *MuDR* remains active (spotted kernels) similar to (A).
(0.57 MB TIF)

Figure S3 Diagrams of LTRs assayed in this study. Schematics of the LTR retrotransposons (black boxes = Long Terminal Repeats ; white boxes = protein coding regions) used in both RT- and qRT-PCR analysis with the region amplified by the primers used in this study underlined with an arrow. The direction of the arrow indicates the orientation of transcripts termed “sense” in the study while transcripts in the opposite orientation were termed “antisense.”
(0.04 MB TIF)

Figure S4 Strand-specific RT-PCR of *rdr2* material. (A,B) Comparison of of RT-PCR products recovered with strand-specific primers in the B73 inbred background (A) and the non-standard *rdr2* mutant background (B). (C) Change in relative abundance (±2 s.e.m.) of *CRM* transcript in *rdr2* mutants as compared to non-mutants by qRT-PCR.
(0.44 MB TIF)

Figure S5 qRT-PCR analysis assaying the enrichment of *Aat*. Enrichment of *Aat*, a polyadenylated Pol II-derived transcript, relative to maize *45S* precursor transcript, which is non-polyadenylated, for the *rpd1* mutant and non-mutant sibling samples used in Figure 5A and 5B.
(2.74 MB TIF)

Figure S6 Dot plot mapping positions of *Arabidopsis*-maize homologous gene pairs. Dot plot mapping positions of homologous gene pairs identified by BLASTP searches for the *Zea mays* chromosome *1* contig compared to (A) *Arabidopsis* chromosome *1*, (B) chromosome *2*, (C) chromosome *4*. Axis numbers indicate the position in the given chromosomal region and circled dots indicate the homologous gene pairs used to define the chromosomal regions referenced in Figure 6 and Table 2.
(0.17 MB TIF)

Figures S7 Gene structure, small RNA profile, and RT-PCR analysis of selected maize-*Arabidopsis* homologs identified in Figure 6 and Figure S6. (A,B) show the structures of the presumed homologs (highlighted in red). A sliding 50 bp window was used to visualize the published small RNA profiles (>22 nt; [2]) for both non-mutants and *mop1-1* homozygotes. Small RNA sequences from the plus strand are above the x-axis and those from the minus stand are below the x-axis. Red bars indicate windows in which the relative number of small RNA hits exceeded the given scale. The repetitive sequence bar details areas of the gene region with similarity to known repetitive sequences identified by the Repbase CENSOR algorithm. DNA transposons are shown in yellow, LTR retrotransposons are shown in blue, and non-LTR retrotransposons in purple. (C) RT-PCR analysis of the putative homologs (with the exception of ZM_chr01_CG_01480 which gave no product) from the non-mutant B73 inbred (+) and *rmr1* mutant (−) plants with *Aat* control.
(0.20 MB TIF)

Figures S8 Gene structure, small RNA profile, and RT-PCR analysis of selected maize-*Arabidopsis* homologs identified in Figure 6 and Figure S6. Analogous analysis as presented in Figure S7.
(3.48 MB TIF)

Figures S9 Gene structure, small RNA profile, and RT-PCR analysis of selected maize-*Arabidopsis* homologs identified in Figure 6 and Figure S6. Analogous analysis as presented in Figure S7.
(0.21 MB TIF)

Figure S10 Flowering time and plant height between *rmr1* mutants and non-mutants. (A) Comparison of flowering times and plant heights between *rmr1-2* homozygotes (*rmr1-2*) and heterozygous siblings (+) (±1 s.e.m.). (B) Analogous measurements for sibling *rmr1-3* genotypes.
(0.05 MB TIF)

Figure S11 UV light microscopy of DAPI-stained nuclei isolated from growing root tips. Comparison of (A) *rmr1-1* heterozygotes and (B) *rmr1-1* homozygous mutants.
(2.00 MB TIF)

Table S1 Relative gene and repetitive element content of *Zea mays* (*Zm*) and *Arabidopsis thaliana* (*At*) chromosomal regions containing syntenous gene pairs.
(0.05 MB DOC)

Table S2 Comparison of the relative number of small RNA sequence tags remaining in *rdr2* (*mop1-1*) mutants relative to the non-mutant genome that are homologous to the attendant repetitive sequence identified in Table S1.
(0.03 MB DOC)

Table S3 Probes and DNA oligonucleotide primers used in this study.
(0.05 MB DOC)

Text S1 Supplemental methods.
(0.05 MB DOC)
