# Supplementary figures and images for: Production and Processing of siRNA Precursor Transcripts from the Highly Repetitive Maize Genome
Source: PLoS Genet. 2009 Aug 14;5(8):e1000598. doi: 10.1371/journal.pgen.1000598 (PMC2725412; doi:10.1371/journal.pgen.1000598)

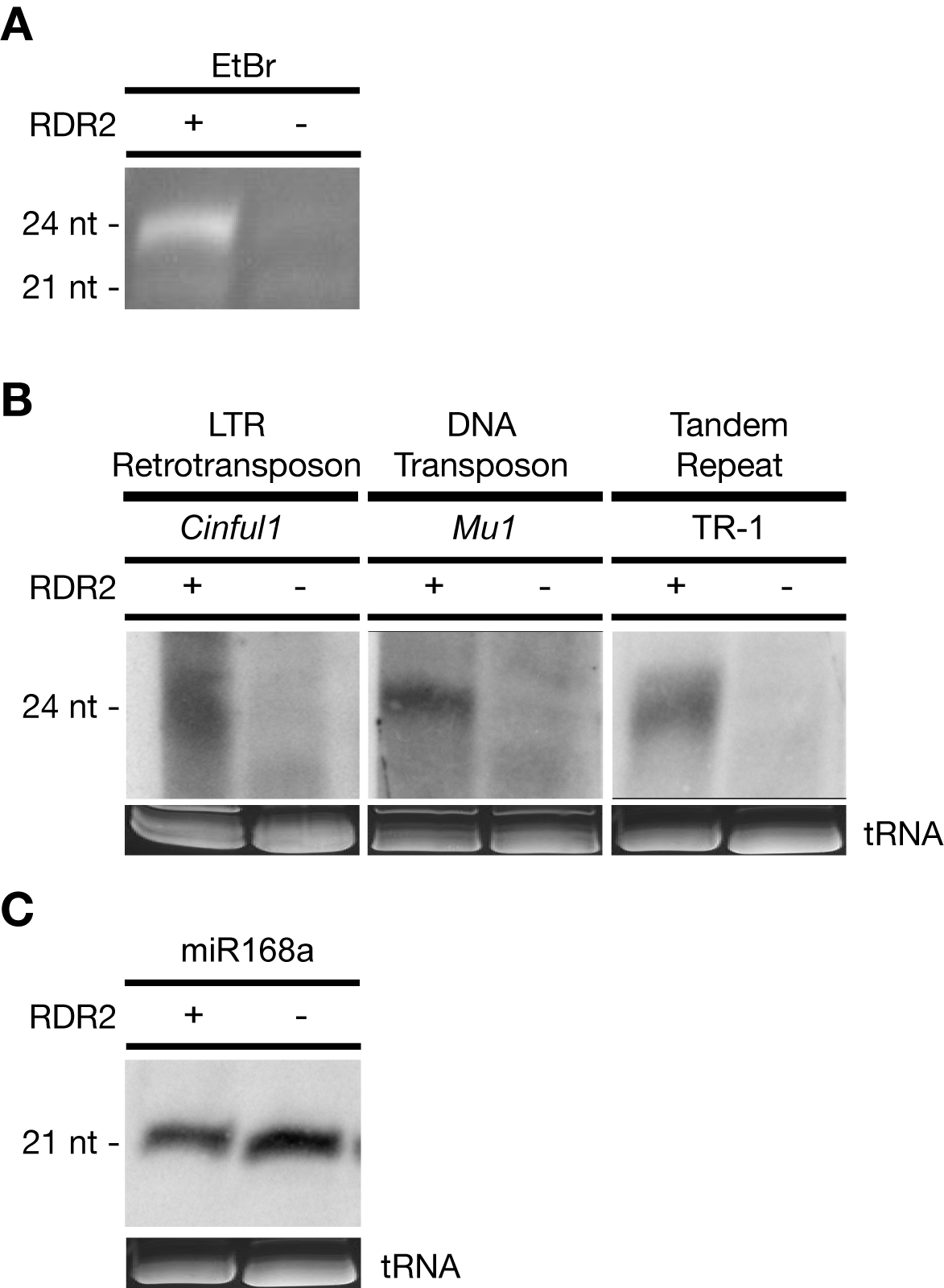

Supplement: Figure S1 — Plants deficient for RDR2 show analogous effects on small RNA populations as rmr1 mutants. (A) EtBr staining of PAGE-separated small RNA fractions from rdr2 mutants (−) and heterozygous siblings (+). (B,C) Small RNA northern blots hybridized with radiolabeled probes of various repetitive maize features and miR168 showing rdr2 mutants specifically lose 24 nt small RNAs corresponding to repetitive sequence. (0.32 MB TIF) [file pgen.1000598.s001.tif]

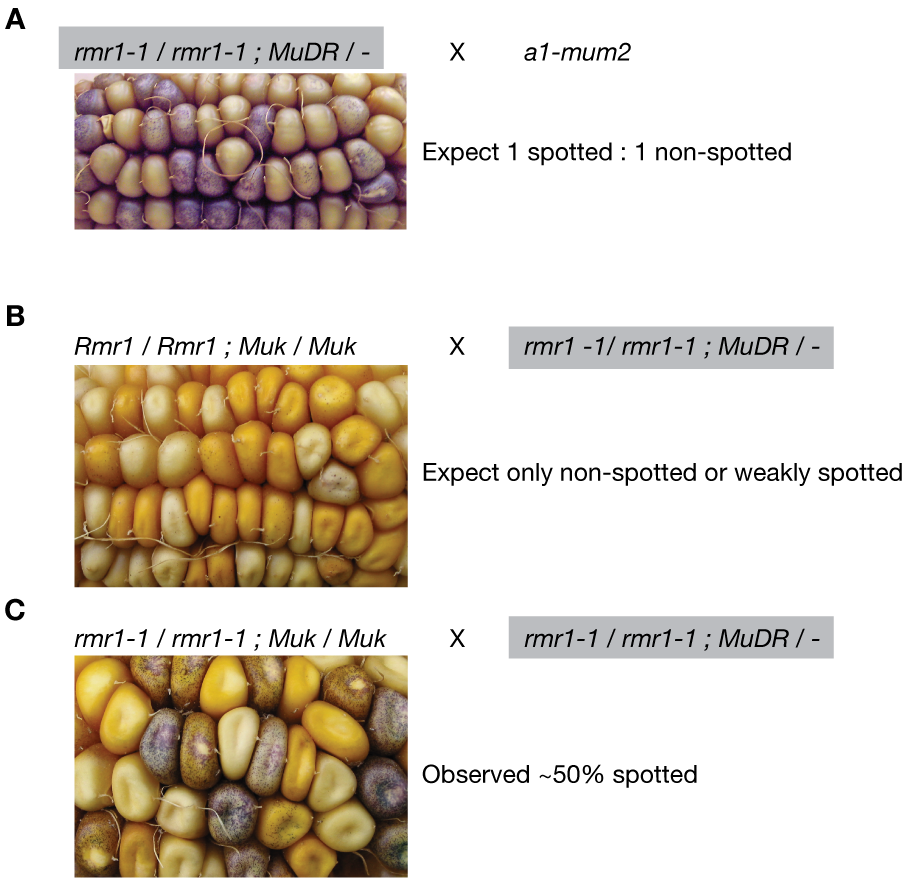

Supplement: Figure S2 — Representative ear progenies displaying active and silenced MuDR functions. (A) Test cross progeny of an rmr1-1 homozygote with an active MuDR element (grey box) by a plant homozygous for the a1-mum2 reporter allele showing active MuDR (spotted kernels) segregates as expected from rmr1 mutants. (B) Cross of parental rmr1-1 homozygote from (A - grey box) to a Muk homozygote illustrating effective silencing of MuDR element. (C) Cross of parental rmr1-1 homozygote from (A- gray box) to an rmr1-1/rmr1-1 ; Muk plant showing MuDR remains active (spotted kernels) similar to (A). (0.57 MB TIF) [file pgen.1000598.s002.tif]

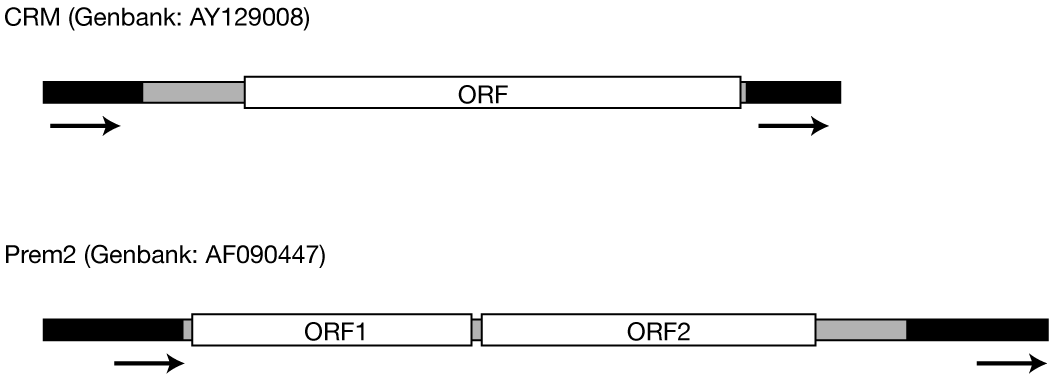

Supplement: Figure S3 — Diagrams of LTRs assayed in this study. Schematics of the LTR retrotransposons (black boxes = Long Terminal Repeats ; white boxes = protein coding regions) used in both RT- and qRT-PCR analysis with the region amplified by the primers used in this study underlined with an arrow. The direction of the arrow indicates the orientation of transcripts termed “sense” in the study while transcripts in the opposite orientation were termed “antisense.” (0.04 MB TIF) [file pgen.1000598.s003.tif]

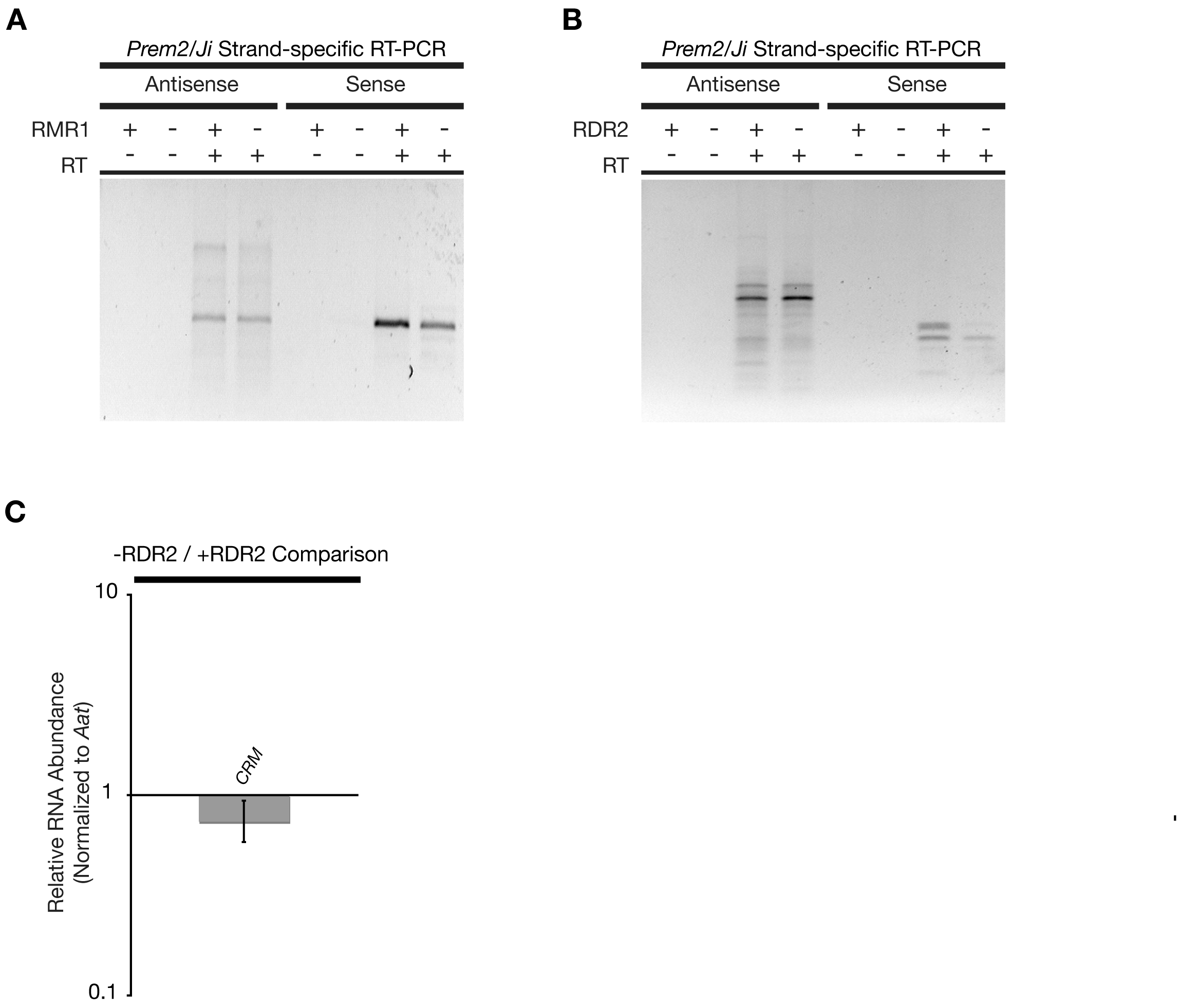

Supplement: Figure S4 — Strand-specific RT-PCR of rdr2 material. (A,B) Comparison of of RT-PCR products recovered with strand-specific primers in the B73 inbred background (A) and the non-standard rdr2 mutant background (B). (C) Change in relative abundance (±2 s.e.m.) of CRM transcript in rdr2 mutants as compared to non-mutants by qRT-PCR. (0.44 MB TIF) [file pgen.1000598.s004.tif]

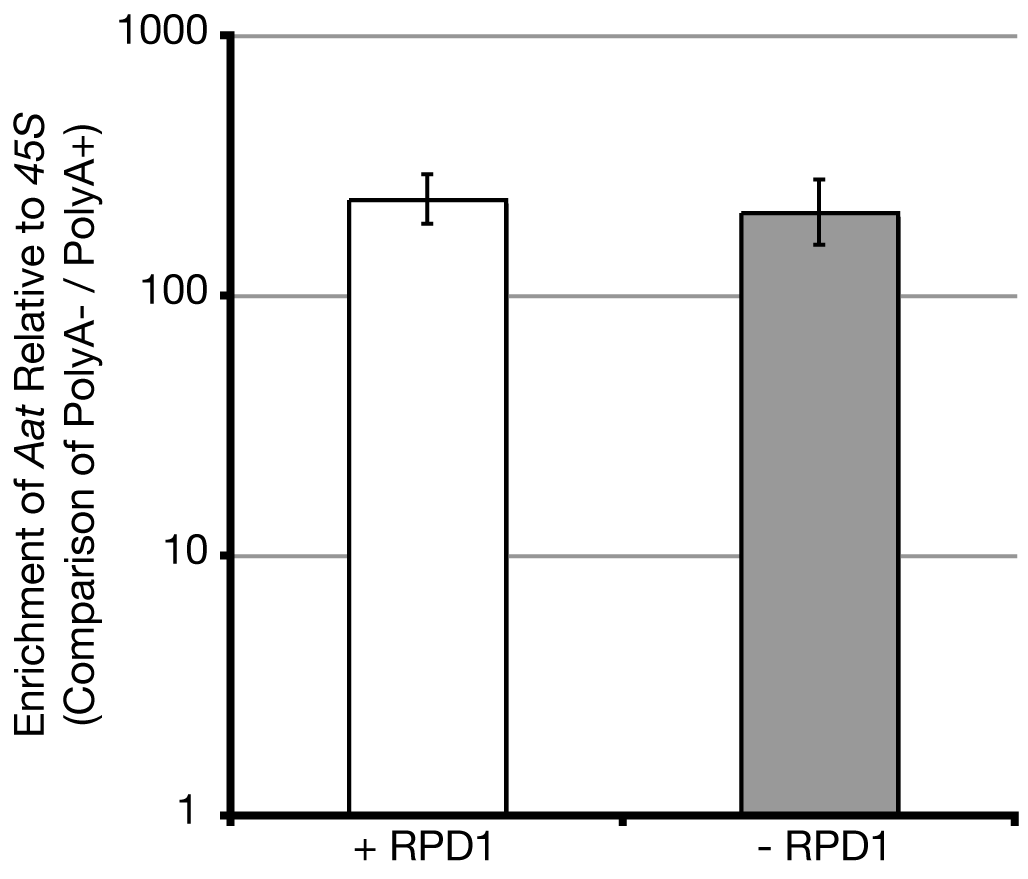

Supplement: Figure S5 — qRT-PCR analysis assaying the enrichment of Aat. Enrichment of Aat, a polyadenylated Pol II-derived transcript, relative to maize 45S precursor transcript, which is non-polyadenylated, for the rpd1 mutant and non-mutant sibling samples used in Figure 5A and 5B. (2.74 MB TIF) [file pgen.1000598.s005.tif]

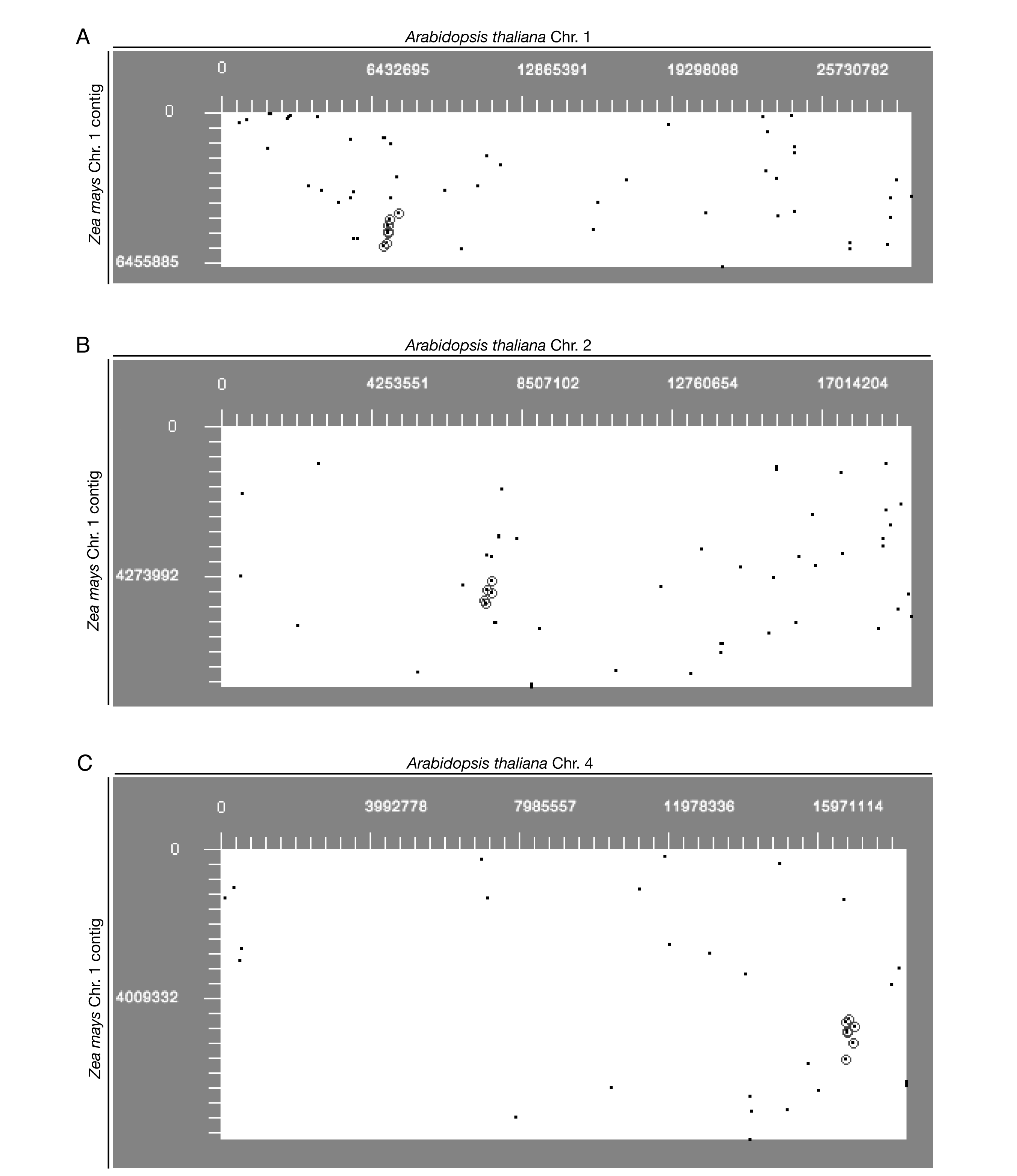

Supplement: Figure S6 — Dot plot mapping positions of Arabidopsis-maize homologous gene pairs. Dot plot mapping positions of homologous gene pairs identified by BLASTP searches for the Zea mays chromosome 1 contig compared to (A) Arabidopsis chromosome 1, (B) chromosome 2, (C) chromosome 4. Axis numbers indicate the position in the given chromosomal region and circled dots indicate the homologous gene pairs used to define the chromosomal regions referenced in Figure 6 and Table 2. (0.17 MB TIF) [file pgen.1000598.s006.tif]

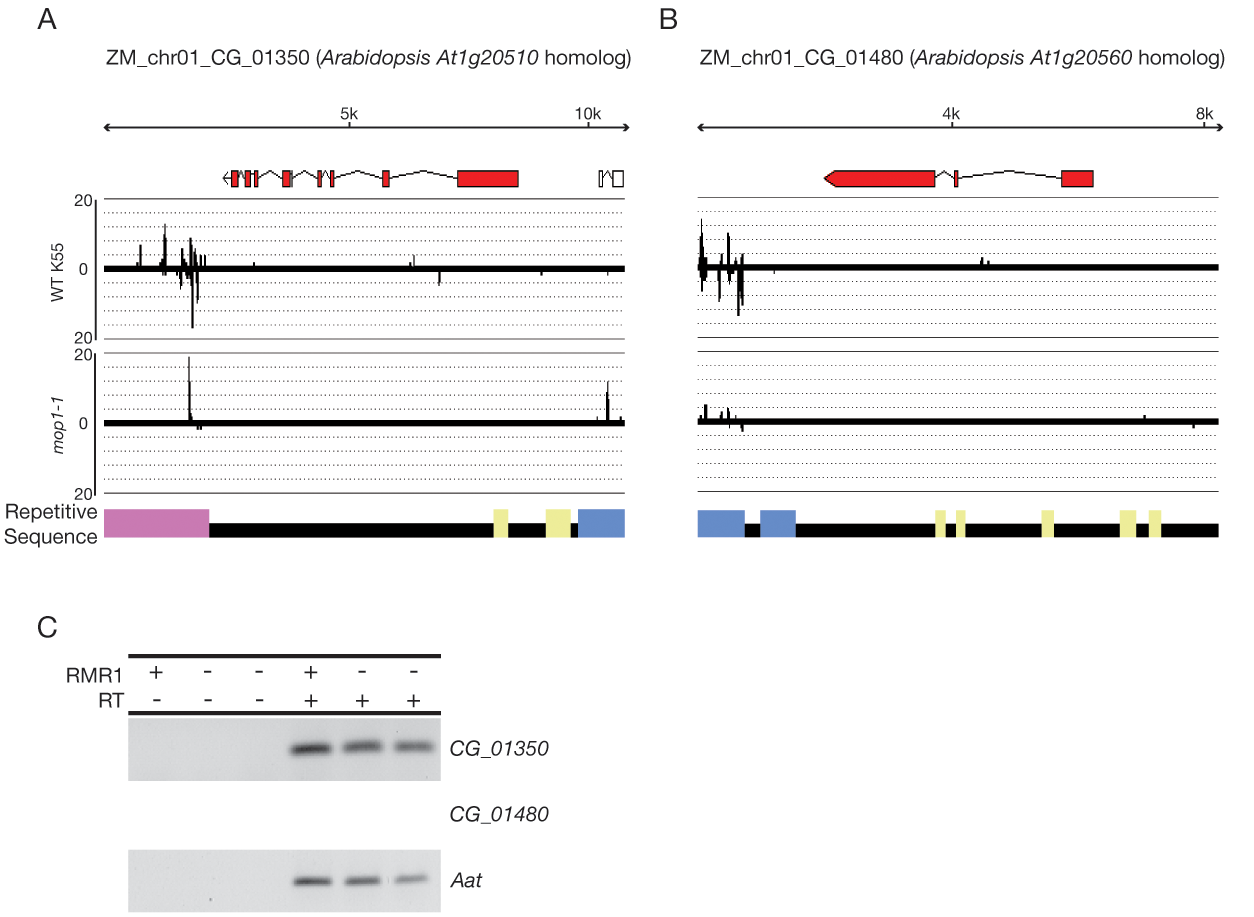

Supplement: Figures S7 — Gene structure, small RNA profile, and RT-PCR analysis of selected maize-Arabidopsis homologs identified in Figure 6 and Figure S6. (A,B) show the structures of the presumed homologs (highlighted in red). A sliding 50 bp window was used to visualize the published small RNA profiles (>22 nt; [2]) for both non-mutants and mop1-1 homozygotes. Small RNA sequences from the plus strand are above the x-axis and those from the minus stand are below the x-axis. Red bars indicate windows in which the relative number of small RNA hits exceeded the given scale. The repetitive sequence bar details areas of the gene region with similarity to known repetitive sequences identified by the Repbase CENSOR algorithm. DNA transposons are shown in yellow, LTR retrotransposons are shown in blue, and non-LTR retrotransposons in purple. (C) RT-PCR analysis of the putative homologs (with the exception of ZM_chr01_CG_01480 which gave no product) from the non-mutant B73 inbred (+) and rmr1 mutant (−) plants with Aat control. (0.20 MB TIF) [file pgen.1000598.s007.tif]

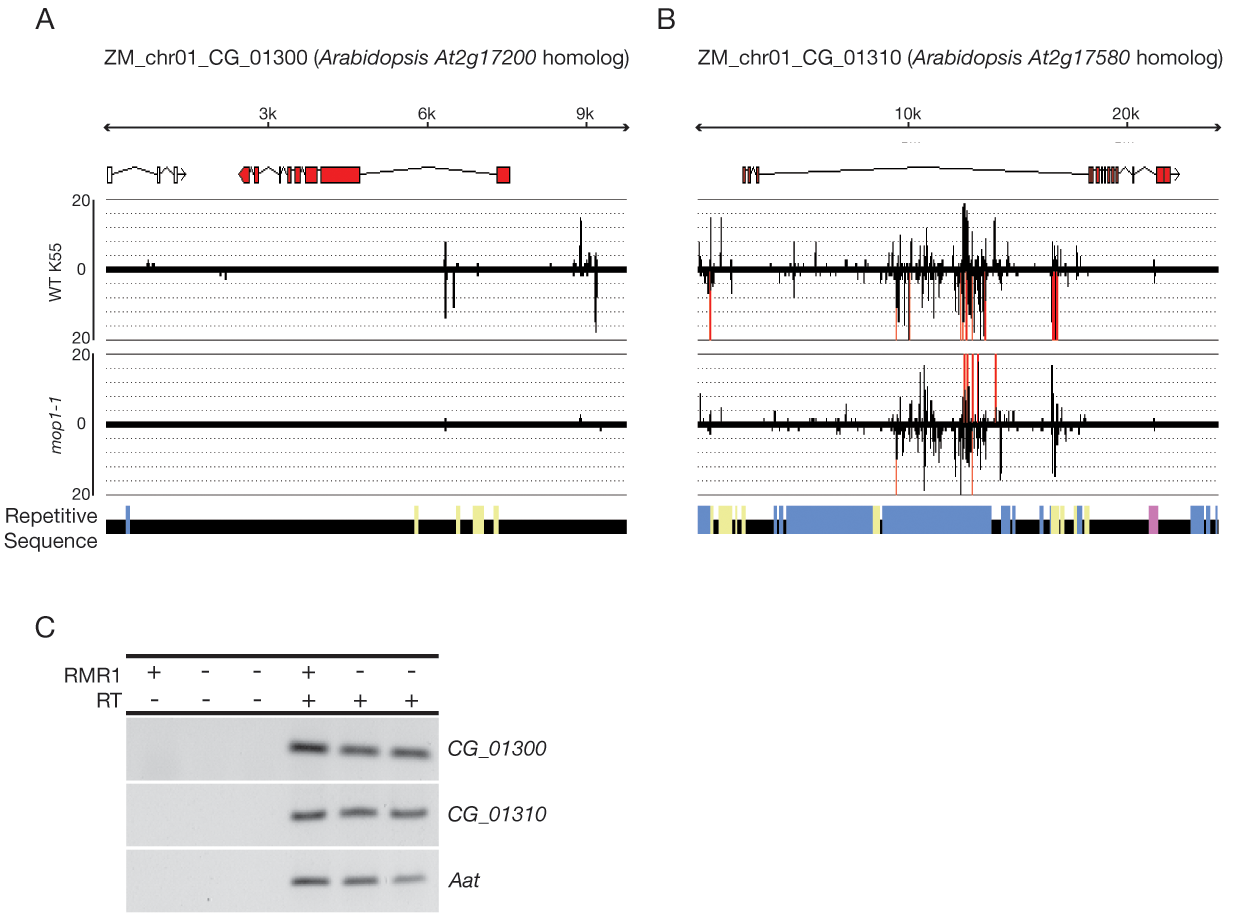

Supplement: Figures S8 — Gene structure, small RNA profile, and RT-PCR analysis of selected maize-Arabidopsis homologs identified in Figure 6 and Figure S6. Analogous analysis as presented in Figure S7. (3.48 MB TIF) [file pgen.1000598.s008.tif]

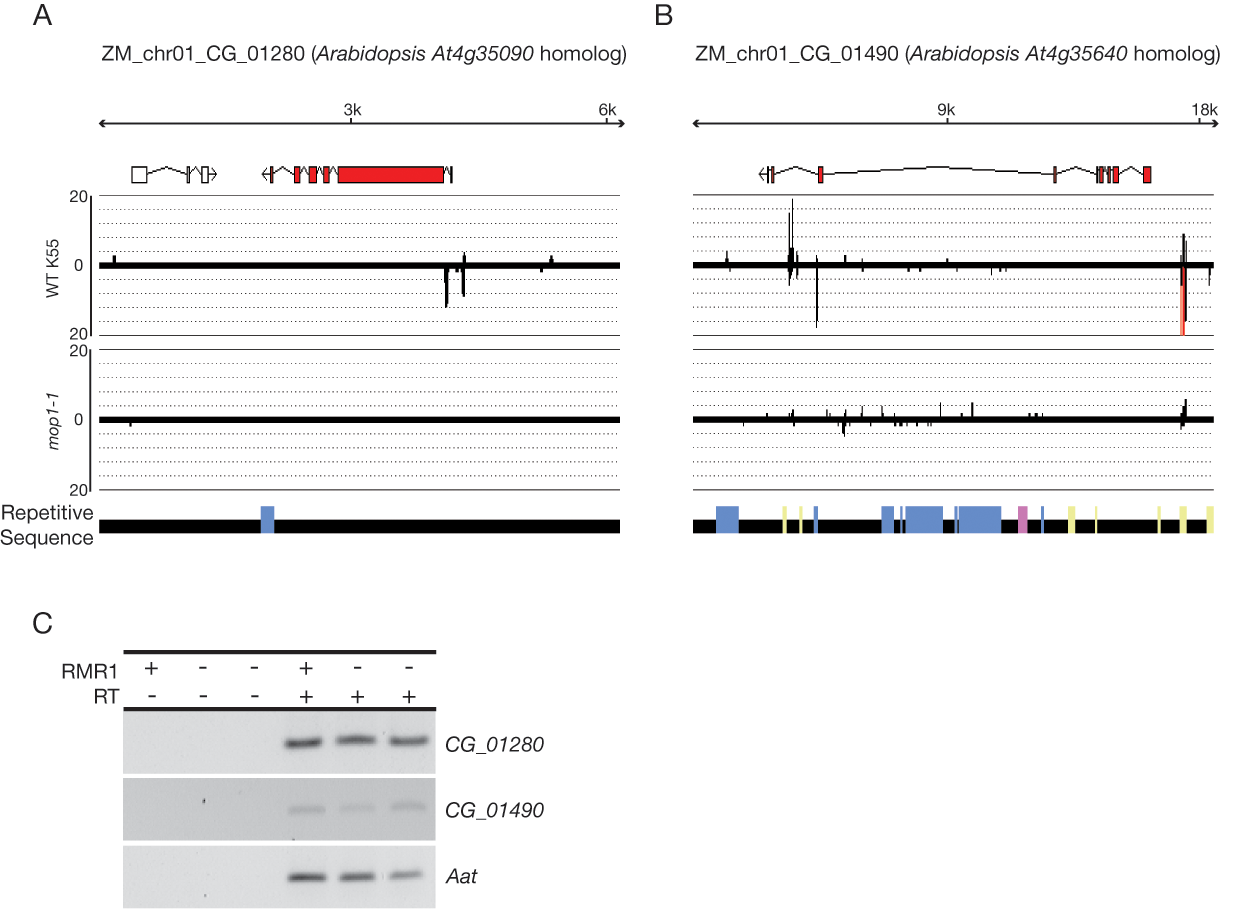

Supplement: Figures S9 — Gene structure, small RNA profile, and RT-PCR analysis of selected maize-Arabidopsis homologs identified in Figure 6 and Figure S6. Analogous analysis as presented in Figure S7. (0.21 MB TIF) [file pgen.1000598.s009.tif]

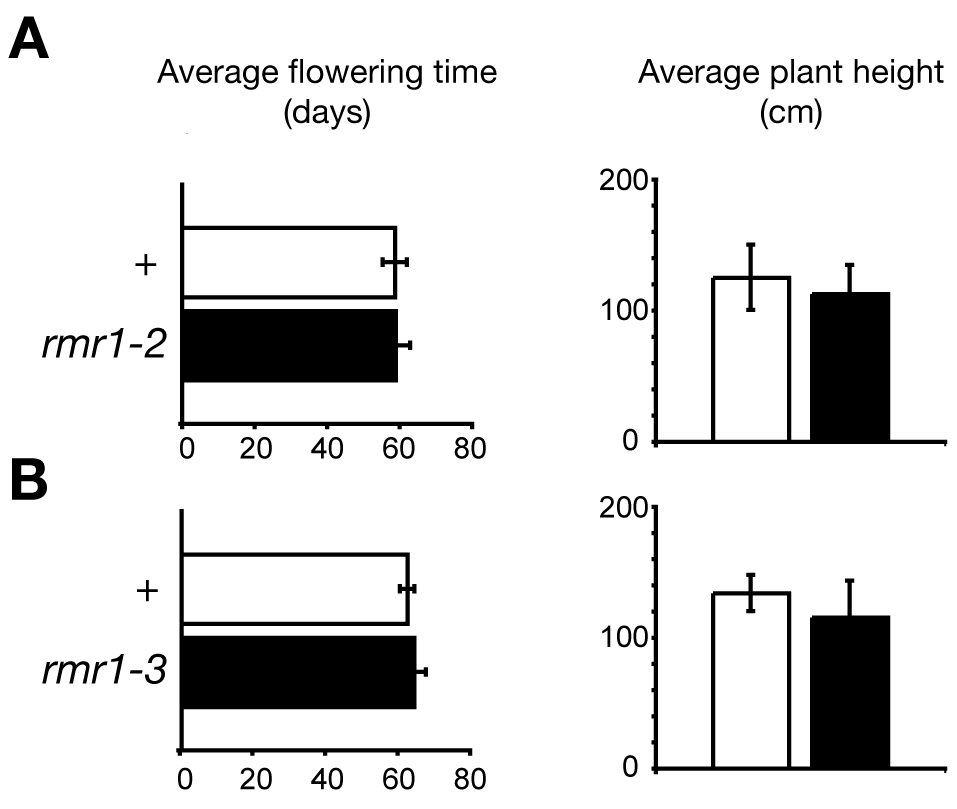

Supplement: Figure S10 — Flowering time and plant height between rmr1 mutants and non-mutants. (A) Comparison of flowering times and plant heights between rmr1-2 homozygotes (rmr1-2) and heterozygous siblings (+) (±1 s.e.m.). (B) Analogous measurements for sibling rmr1-3 genotypes. (0.05 MB TIF) [file pgen.1000598.s010.tif]

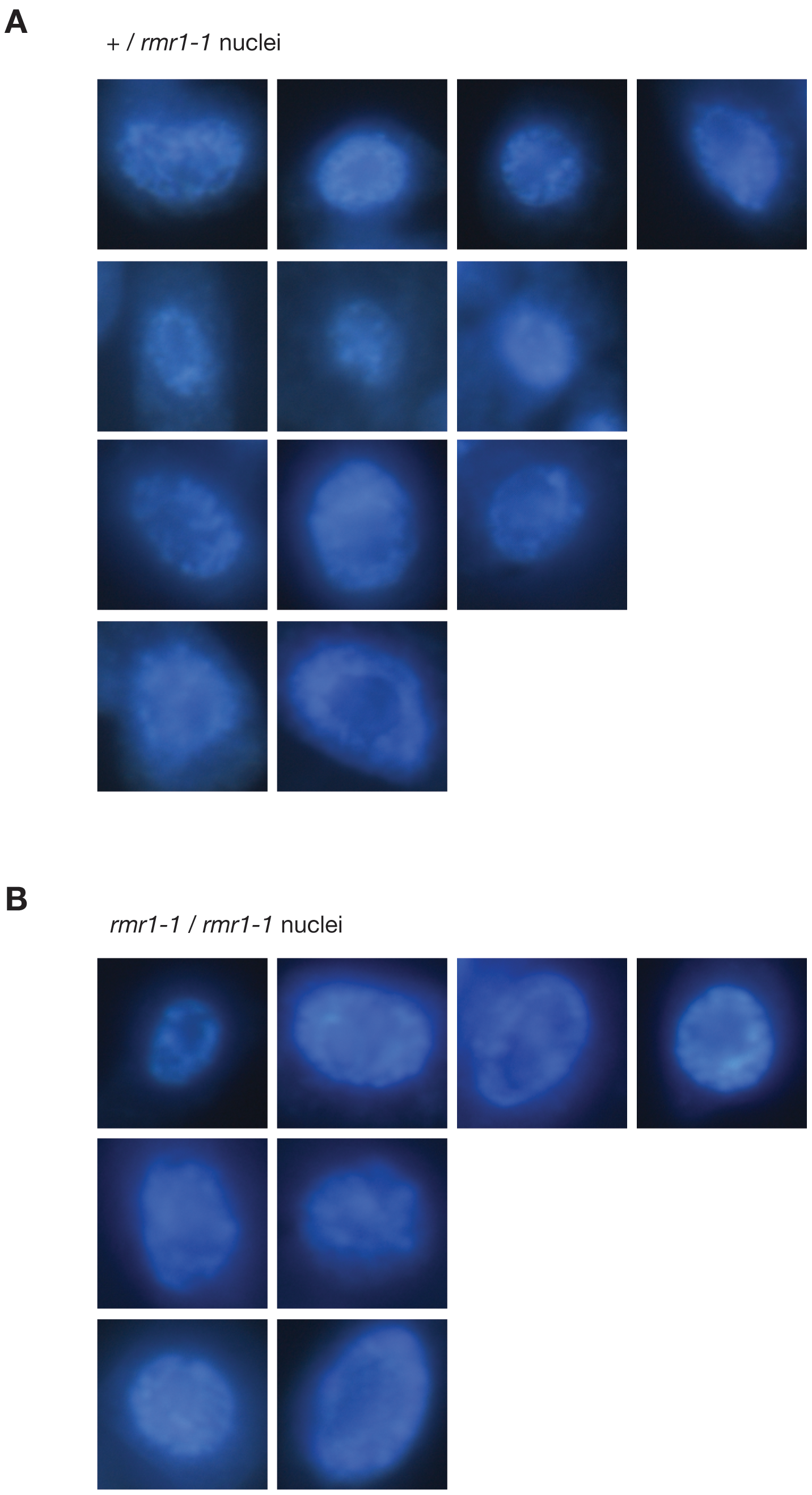

Supplement: Figure S11 — UV light microscopy of DAPI-stained nuclei isolated from growing root tips. Comparison of (A) rmr1-1 heterozygotes and (B) rmr1-1 homozygous mutants. (2.00 MB TIF) [file pgen.1000598.s011.tif]
