# Supplementary material for: Production and Processing of siRNA Precursor Transcripts from the Highly Repetitive Maize Genome
Source: PLoS Genet. 2009 Aug 14;5(8):e1000598. doi: 10.1371/journal.pgen.1000598 (PMC2725412; doi:10.1371/journal.pgen.1000598)
Supplement: Table S1 — Relative gene and repetitive element content of Zea mays (Zm) and Arabidopsis thaliana (At) chromosomal regions containing syntenous gene pairs. (0.05 MB DOC) [file pgen.1000598.s012.doc]

| **Chromosomal Region^1^** | | |  | **Percent of specific transposon classes** | | |
| --- | --- | --- | --- | --- | --- | --- |
|  |  |  | *Gene Density (Gene/kb)* | *DNA Transposon* | *LTR retro-transposon* | *non-LTR retro-transposon* |
|  | *Start* | *Stop* |  |  |  |  |
|  |  |  |  |  |  |  |
| *Zm* chr1 contig |  |  |  |  |  |  |
|  | 4300000 | 5800000 | 0.042 | 3.97% | 58.26% | 2.03% |
|  |  |  |  |  |  |  |
| *At* chr1 | 6900000 | 7600000 | 0.253 | 3.34% | 0.71% | 0.13% |
| *At* chr2 | 7400000 | 7700000 | 0.243 | 4.72% | 3.18% | 0.41% |
| *At* chr4 | 16650000 | 16950000 | 0.283 | 1.06% | 0.10% | 0.00% |
|  |  |  |  |  |  |  |

^1^Start and stop values for maize chromosome *1* are relative to the start of the chromosome *1* contig as described [Supplemental Reference 1].
