# Supplementary material for: Production and Processing of siRNA Precursor Transcripts from the Highly Repetitive Maize Genome
Source: PLoS Genet. 2009 Aug 14;5(8):e1000598. doi: 10.1371/journal.pgen.1000598 (PMC2725412; doi:10.1371/journal.pgen.1000598)
Supplement: Table S2 — Comparison of the relative number of small RNA sequence tags remaining in rdr2 (mop1-1) mutants relative to the non-mutant genome that are homologous to the attendant repetitive sequence identified in Table S1. (0.03 MB DOC) [file pgen.1000598.s013.doc]

|  |  | **Percent of *mop1-1* sequence tags compared to wild type** | | |
| --- | --- | --- | --- | --- |
| *Chromosomal Region* | | *500 bp upstream* | *Intragenic* | *500 bp downstream* |
| Zm chromosome 1 contig (syntenous region) | | 5.70% | 9.04% | 14.86% |
|  |  |  |  |  |
| Zm chromosome 1 contig (complete) | | 6.83% | 28.15% | 8.62% |
| Zm chromosome 9 contig (complete) | | 9.54% | 36.26% | 50.53% |
|  | |  |  |  |

The syntenous regions refer to the chromosomal and contig regions identified in Figure 6 and Table S1. The complete contig refers to the sequence generated by [Supplemental Reference 1].
