# Supplementary material for: Production and Processing of siRNA Precursor Transcripts from the Highly Repetitive Maize Genome
Source: PLoS Genet. 2009 Aug 14;5(8):e1000598. doi: 10.1371/journal.pgen.1000598 (PMC2725412; doi:10.1371/journal.pgen.1000598)
Supplement: Table S3 — Probes and DNA oligonucleotide primers used in this study. (0.05 MB DOC) [file pgen.1000598.s014.doc]

| **Probe used** | **Figure** | **Generated** | **Reference** |
| --- | --- | --- | --- |
| Prem2/Ji | Figure 1 | riboprobe generated from T/A clone of PCR product from previously described primers | Lamb et a., 2007 |
| Cinful1 | Figure 1, S1 | riboprobe generated from T/A clone of PCR product from previously described primers | Lamb et a., 2007 |
| CentA | Figure 1 | riboprobe generated from T/A clone of PCR product from previously described primers | Lamb et a., 2007 |
| CRM | Figure 1 | riboprobe generated from T/A clone of PCR product from previously described primers | Lamb et a., 2007 |
| Mu1 | Figure 1, S1 | riboprobe generated from previously described clone | Woodhouse et al., 2006 |
| TR1 | Figure 1, S1 | riboprobe generated from PCR product from TR1-358-F (5 ' - GAG GGC ATT GTA TTC ACA CG) and TR1-358-R (5 ' - TGG ACA GTT CTC TCA CGC AAT) primers | Ananiev et al., 1998 |
| miR168 | Figure 1, S1 | Endlabled DNA oligo (5 ' - GTC CCG ATC TGC ACC AAG CGA) identical to miR168* | Chuck et al., 2007 |
| Mu1 | Figure 2 | Klenow labled DNA fragement from Mu1 clone | Lisch et al., 2002 |
| 45S | Figure S5 | 45S precursor transcript amplified using Primers 45S.4 ( 5 ' - TTG AGA AGT GCT TGC GTG C) and -45S.5 ( 5 ' - GTA GCA CGT CCT CGC AGA C) |  |
