# Supplementary material for: Production and Processing of siRNA Precursor Transcripts from the Highly Repetitive Maize Genome
Source: PLoS Genet. 2009 Aug 14;5(8):e1000598. doi: 10.1371/journal.pgen.1000598 (PMC2725412; doi:10.1371/journal.pgen.1000598)
Supplement: Text S1 — Supplemental methods. (0.05 MB DOC) [file pgen.1000598.s015.doc]

**Supplemental Materials and Methods**

Growth conditions for genetic stocks used

Materials used for small RNA analyses were grown at the Oxford Tract field facility in Berkeley, CA. Materials used for Southern blots were grown at the Gill Tract field facility in Albany, CA. Materials used for RT-PCR analysis and cytological analysis were germinated on damp paper pads in Petri dishes following seed surface sterilization with 50% bleach. Materials for morphometric analyses in Figures 7A, and S10 were grown in Hanapepe, HI. The materials for the recombinant inbred line analysis in Figure 7C were grown in multiple locations with S_5_ measurements carried out at the Gill Tract and Oxford tract field facilities.

*rdr2* (*mop1-1*) small RNA analysis

All blots and ethidium bromide staining in Figure S1were carried out as described for the *rmr1* small RNA analysis.

UV light microscopy

The nuclei in Figure S11 were isolated and mounted as described in the main text. DAPI-stained nuclei were then visualized on a Zeiss Axioplan 2 UV microscope with a Axiocam digital camera (Zeiss).

Analysis of selected *Zea mays* gene models

Selected maize gene models in Figures S7-S9 from the chromosome *1* contig [1] illustrated in Figure 6 and listed in Table S1 were used as BLAST queries against the B73 and *rdr2* (*mop1-1*) small RNA databases [2] looking for exact matches >22nt. These databases return relative numbers of small RNA sequencing reads for each of the K55 and *rdr2* (*mop1-1*) genotypes. These relative numbers of small RNA signatures were plotted across the given locus using a sliding 50 bp window and a custom Perl script. These loci were also analyzed with the CENSOR algorithm to identify repetitive sequences [3].

RT-PCR of selected *Zea mays* gene models

RT-PCR was carried out as described in the main text comparing wild-type B73 (97% B73) plants and *rmr1* mutant plants derived from *rmr1-1* introgressions into B73 (94% B73). Primers used are listed in Table S3.

Bioinformatic analysis of attendant repetitive sequences.

To generate the data in Table S2, the repetitive sequences identified in Table 1 were used to query the K55 inbred and *rdr2* (*mop1-1*) small RNA populations using BLAST. Using only exact matches with lengths >22nt, the relative percent of small RNA signal remaining in *rdr2* mutants was calculated by dividing the total number of reads found in the non-mutant K55 inbred by the total number in the *rdr2* mutant population as shown in Table S2.

**References**

1. Bruggmann R, Bharti AK, Gundlach H, Lai J, Young S, et al. (2006) Uneven chromosome contraction and expansion in the maize genome. Genome Res 16: 1241-1251.

2. Nobuta K, Lu C, Shrivastava R, Pillay M, De Paoli E, et al. (2008) Distinct size distribution of endogeneous siRNAs in maize: Evidence from deep sequencing in the mop1-1 mutant. Proc Natl Acad Sci U S A 105: 14958-14963.

3. Kohany O, Gentles AJ, Hankus L, Jurka J (2006) Annotation, submission and screening of repetitive elements in Repbase: RepbaseSubmitter and Censor. BMC Bioinformatics 7: 474.
